# Spike Generators and Cell Signaling in the Human Auditory Nerve: An Ultrastructural, Super-Resolution, and Gene Hybridization Study

**DOI:** 10.3389/fncel.2021.642211

**Published:** 2021-03-16

**Authors:** Wei Liu, Maria Luque, Hao Li, Anneliese Schrott-Fischer, Rudolf Glueckert, Sven Tylstedt, Gunesh Rajan, Hanif Ladak, Sumit Agrawal, Helge Rask-Andersen

**Affiliations:** ^1^Section of Otolaryngology, Department of Surgical Sciences, Head and Neck Surgery, Uppsala University Hospital, Uppsala, Sweden; ^2^Department of Otorhinolaryngology, Medical University of Innsbruck, Innsbruck, Austria; ^3^Department of Olaryngology, Västerviks Hospital, Västervik, Sweden; ^4^Department of Otolaryngology, Head & Neck Surgery, Luzerner Kantonsspital, Luzern, Switzerland; ^5^Department of Otolaryngology, Head & Neck Surgery, Division of Surgery, Medical School, University of Western Australia, Perth, WA, Australia; ^6^Department of Otolaryngology-Head and Neck Surgery, Department of Medical Biophysics and Department of Electrical and Computer Engineering, Western University, London, ON, Canada; ^7^Department of Otolaryngology-Head and Neck Surgery, Western University, London, ON, Canada

**Keywords:** human, auditory nerve, gene expression, structured illumination microscopy, spike generation

## Abstract

**Background:** The human auditory nerve contains 30,000 nerve fibers (NFs) that relay complex speech information to the brain with spectacular acuity. How speech is coded and influenced by various conditions is not known. It is also uncertain whether human nerve signaling involves exclusive proteins and gene manifestations compared with that of other species. Such information is difficult to determine due to the vulnerable, “esoteric,” and encapsulated human ear surrounded by the hardest bone in the body. We collected human inner ear material for nanoscale visualization combining transmission electron microscopy (TEM), super-resolution structured illumination microscopy (SR-SIM), and RNA-scope analysis for the first time. Our aim was to gain information about the molecular instruments in human auditory nerve processing and deviations, and ways to perform electric modeling of prosthetic devices.

**Material and Methods:** Human tissue was collected during trans-cochlear procedures to remove petro-clival meningioma after ethical permission. Cochlear neurons were processed for electron microscopy, confocal microscopy (CM), SR-SIM, and high-sensitive *in situ* hybridization for labeling single mRNA transcripts to detect ion channel and transporter proteins associated with nerve signal initiation and conductance.

**Results:** Transport proteins and RNA transcripts were localized at the subcellular level. Hemi-nodal proteins were identified beneath the inner hair cells (IHCs). Voltage-gated ion channels (VGICs) were expressed in the spiral ganglion (SG) and axonal initial segments (AISs). Nodes of Ranvier (NR) expressed Nav1.6 proteins, and encoding genes critical for inter-cellular coupling were disclosed.

**Discussion:** Our results suggest that initial spike generators are located beneath the IHCs in humans. The first NRs appear at different places. Additional spike generators and transcellular communication may boost, sharpen, and synchronize afferent signals by cell clusters at different frequency bands. These instruments may be essential for the filtering of complex sounds and may be challenged by various pathological conditions.

## Introduction

### Human Speech—Reception and Spike Generation

Humans have developed sophisticated abilities to produce and perceive oral speech. This involves particular anatomy, complex neural circuits in the brain, and a perceptual apparatus that deciphers “multifaceted” air-borne signals (Hockett et al., 1964). How this cladistics took place is fiercely discussed among linguistic anthropologists. Its components, such as morphology, phonetics, and semantics, may have been shaped by several environmental factors (Wiener, 1984). In all cases, the human auditory nerve relays intricate speech-coded information to the brain that depends on an unbroken signal acuity to the central nervous system (CNS). The established signals are vulnerable, and their conservation is essential for proper decrypting. They are not readily restored centrally once distorted by tumor compression or deficient conversion at the inner hair cell (IHC) ribbon synapse. Gene mutations (FOXP2) have been associated with abnormal development of neural structures important for human speech and language (Lai et al., 2001), and the locus on chromosome 16 has been associated with specific language impairment (Newbury et al., 2005), a more or less central deficiency in perception of speech (Bishop et al., 2007).

It remains unclear how speech is coded in the auditory nerve, but it has been studied in animal models (Kiang, 1980, Khanna and Teich, 1989). Even though potentials recorded from the cochlea and auditory nerve are similar for most mammals, different species have developed arrangements to optimally process sound most relevant for their survival (Theunissen and Elie, 2014). Evolutionary adaptation may include modifications of inherent molecular systems. Since there are substantial anatomical differences between humans and other species (Kimura et al., 1979; Ota and Kimura, 1980; Arnold, 1987; Spoendlin and Schrott, 1988; Tylstedt and Rask-Andersen, 2001; Liu et al., 2015), distinct features may have developed and been reflected in the morphology, distribution of coding proteins, excitation pattern, and nerve conductivity. Researchers have indicated that frequency resolution relevant for speech development is higher in humans than in laboratory animals (Shera et al., 2010; Sumner et al., 2018). Nonetheless, this remains controversial (Ruggero and Temchin, 2005; Lopez-Poveda and Eustaquio-Martin, 2013), and studies have claimed that sharpness of tuning is similar in all mammals and birds.

It is undetermined how and where action potentials (APs) are generated in the human auditory nerve. Possible sites are the (1) nerve-receptor junction, (2) spiral ganglion (SG), (3) axonal initial segments (AISs), and (4) Nodes of Ranvier (NR). Studies of voltage-gated ion channels (VGICs) were performed in several non-human species with variable results (Mo and Davis, 1997; Adamson et al., 2002; Hossain et al., 2005; Fryatt et al., 2009; McLean et al., 2009; Smith et al., 2015; Kim and Rutherford, 2016). A multitude of voltage-gated K^+^ channels with various gating kinetics were discovered in the auditory pathway (Liu Q, et al., 2014), and literature reviews on these have been presented (Oak and Yi, 2014; Reijntjes and Pyott, 2016). RNA sequencing and single molecule *in situ* hybridization mapped transcripts encoding potassium channels were found to be essential for normal auditory function (Reijntjes et al., 2019). Different K^+^-channels are thought to contribute to individual neuronal coding frequencies in the auditory system (Adamson et al., 2002). Single-cell RNA sequencing demonstrated that type I SG neurons (SGNs) are molecularly diverse and identified three subclasses of type I neurons. They were subdivided into six classes based on the genetic framework defining intensity coding properties in a transcriptional catalog of the murine cochlea (Petitpré et al., 2018; Sun et al., 2018). Surprisingly, disruption of IHC signaling before hearing onset was found to influence spontaneous activity and molecular diversification of type I cells (SGNs) (Sun et al., 2018).

A remarkable outcome of speech recognition is gained in the severely hearing impaired by today's auditory electric prostheses, even in patients lacking peripheral dendrites. This suggests that electrically evoked speech signals may be relayed centrally without peripheral or electro-phonic hair cell stimulation. How this happens is virtually unknown.

### Goals of the Present Investigation

We aimed to further analyze and review the micro-anatomy of the human cochlea and auditory nerve using transmission and scanning electron microscopy and 3D imaging. In addition, efforts were made to localize VGICs, their associated proteins and ion transporter Na/K-ATPase and their isoforms using immunohistochemistry and high-resolution structured illumination microscopy (SR-SIM) and confocal microscopy (CM). A first attempt was made to use *in situ* RNA hybridization to detect mRNA transcripts. For this, tissue was harvested in connection with surgeries for life-threatening petro-clival meningioma where the cochlea had to be sacrificed. Ethical permission and patient consent were obtained. Since cochlear function was preserved, it offered unique possibilities to study some of the molecular organization under “near-normal” settings. Besides, we searched for alternate cellular communication pathways capable of synchronized firing that could be essential for processing complex sounds in humans. One donated human temporal bone was analyzed using micro-computerized tomography (MicroCT) and soft tissue staining. Hopefully, the results may bring further elucidation on spike generation and signal characteristics in the human auditory nerve. It may provide information on how and where electric prostheses target stimulation of the human nerve. Due to the limited amount of tissue that can be collected at surgery, a quantitative display and gradient molecular expression of VGICs along the entire cochlear spiral is not possible at this stage.

## Materials and Methods

### Ethical Statements

The study of human cochleae was approved by the local ethics committee (Etikprövningsnämnden Uppsala, no. 99398, 22/9 1999, cont, 2003, no. C254/4; C209/10, no. C45/7 2007, Dnr. 2013/190), and patient consent was obtained. Ethics approval for the microCT project was obtained from the University of Western Australia (UWA, RA/4/1/5210), and the human temporal bones were provided by the Department of Anatomy at UWA. The study adhered to the rules of the Declaration of Helsinki.

### Tissue Sampling

The surgical specimens were from patients suffering from life-threatening posterior cranial fossa meningioma compressing the brain stem (Rask-Andersen et al., 1997). Human cochleae were harvested at major trans-cochlear skull base surgeries, including facial nerve rerouting. The operations were performed at Uppsala University Hospital by a team of neurosurgeons and oto-neuro-surgeons. Five cochleae were dissected out using diamond drills of various sizes (Table 1). Six Dunkin Hartley guinea pigs were processed and underwent similar fixation and immunohistochemistry.

**Table 1:** Patient data and functioning of their cochleae used for IHC and RNA-scope.

**Age**	**Gender**	**PTT/SD**	**Analysis**
43	Female	50 dB (1–8 kHz)	IHC
51	Male	Normal	IHC
72	Male	50 dB (2–4 kHz)	IHC
67	Female	Normal	IHC
67	Female	SD 85%	IHC, RNA-scope

*TEM and SEM data were obtained from archival material as described in papers (Tylstedt et al., 1997; Rask-Andersen et al., 2012; Liu W, et al., 2014). PTT, pure tone thresholds; SD, speech discrimination. The specimens were from persons without any known hearing impairment and were obtained at surgery for removal of large, life-threatening petroclival meningioma where the cochlea hade to be sacrificed during trans-cochlear surgery. Re-routing of the facial nerve is performed routinely at this type of surgery*.

### Immunohistochemistry

Immunohistochemistry procedures on human cochlear sections were described in previous publications (Liu et al., 2009, 2020). In short, tissue was fixed in a solution of 4% (or 2% for sodium channels) paraformaldehyde (PFD) phosphate buffer solution (PBS). Different fixation durations are determined by channel types detected, ranging from 45 min to hours. After fixation, the fixative was replaced with 0.1 M PBS, and cochleae were decalcified in 10% ethylene-diamine-tetra-acetic acid (EDTA) solution at pH 7.2 for 4 weeks. The cochleae were embedded in Tissue-Tek OCT embedding compound (Polysciences, Inc., Warrington, PA, USA), rapidly frozen, and sectioned at 8–10 μm using a cryostat microtome. Sections were incubated with an antibody solution under a humidified atmosphere at 4°C for 20 h. Sections were incubated with secondary antibodies conjugated to Alexa Fluor (Thermo Fisher Scientific, Uppsala) counterstained with the nuclear stain 4′,6-diamidino-2-phenylindole dihydro-chloride (DAPI), mounted with ProLong® Gold Antifade Mountant (Thermo Fisher Scientific, Uppsala, Catalog number: P10144), and then covered with the specified cover glass compatible with both confocal and super-resolution microscopes. Primary and secondary antibody controls and labeling controls were used to exclude endogenous labeling or reaction products (Burry, 2011). The antibodies used for immunohistochemistry are shown in Table 2.

**Table 2:** Antibodies used in the present investigation.

**Antibody**	**Type**	**Host**	**Cat#**	**Company**
ATPase (α1)	Monoclonal (m)	Mouse (m)	NB300-146	Novus
ATPase (α2)	Polyclonal (p)	Rabbit (r)	AP5828c-ev	Nordic BioSite
ATPase (α3)	m	m	MA3-915	Thermo Fisher
ATPase (β1)	m	m	Ma3-930	Abcam
ATPase (β2)	p	r	PA5-26279	Invitrogen
NKCC1	p	r	ab59791	Abcam
Laminin β2	m	r	05-206	Millipore
Cx30	p	r	71-2200	Invitrogen
Cx26	m	m	33-5800	Invitrogen
Cx26	p	r	ACC-2121	Alomone
Cx36	m	m	37-4600	Invitrogen
Cx43	p	r	71-0700	Invitrogen
Cx43	m	m	MAB3068	Millipore
KCa1.1	p	r	APC-107	Alomone
Collagen II	m	m	CP18	Millipore
Collagen IV	p	goat	AB769	Millipore
MBP	m	r	#AB980,	Millipore
S-100	p	r	Z 0311	Dako
Tuj-1	m	m	MAB1637	Millipore
Parvalb.	m	m	MAB1572	Chemicon
Tuj-1	p	r	#04-1049	Millipore
Laminin β2	m	r	#05-206	Millipore
GFAP	p	r	AB5804	Chemicon
GFAP	m	m	MAB360	Millipore
CGRP	p	r	Ab71225	Abcam
Kv1.1	p	r	APC-161	Alomone Labs
Kv1.2	p	r	APC-010	Alomone Labs
Kv3.1	p	r	APC-014	Alomone Labs
Kv7.1	p	r	APC-022	Alomone Labs
Ankyrin G	m	m	NBP2-59310	Novus
Caspr-1	m	m	SMC-370D	Nordic BioSite
Pan-Nav	p	r	ASC-003	Alomone Labs
Nav1.1	m	m	S74-71	Novus
Nav1.3	p	r	ASC-004	Alomone Labs
Nav1.6	m	m	SMC-378D	Nordic BioSite
Nav1.7	p	r	ASC-008	Alomone Labs
Nav1.8	p	r	ASC-016	Alomone Labs
Nav1.9	p	r	ASC-017	Alomone Labs

Stained sections were first investigated with an inverted fluorescence microscope (TE2000; Nikon, Tokyo, Japan) equipped with a spot digital camera with three filters (for emission spectra maxima at 358, 461, and 555 nm). Image-processing software (NIS Element BR-3.2; Nikon), including image merging and a fluorescence intensity analyzer, was installed on a computer system connected to the microscope. For laser CM, we used the same microscope equipped with a three-channel laser emission system. SR-SIM was performed (Gustafsson et al., 2008) using a Zeiss Elyra S.1 SIM system and a 63×/1.4 oil Plan-Apochromat objective (Zeiss, Oberkochen, Germany), sCMOS camera (PCO Edge), and ZEN 2012 software (Carl Zeiss Microscope). The resolution of the SR-SIM system at BioVis, Uppsala University, was 107 nm in the X–Y plane and 394 nm in the Z plane. The following laser and filter setup was as follows: 405 nm laser of excitation coupled with BP 420–480 + LP 750 filter, 488 nm laser of excitation with BP 495–550 + LP750 filter, 561 nm laser of excitation with BP 570–620 + LP 750 filter, and 647 nm laser of excitation with LP 655 filter. From the SR-SIM dataset, 3D reconstruction was done with Imaris 8.2 (Bitplane, Zürich, Switzerland). A bright-field channel was able to merge fluorescence channels to visualize cell/tissue borders.

### RNA-Scope Protocol

Fixed-frozen human tissue sections underwent pretreatment with H_2_O_2_ (10 min, RT) and protease III (30 min, 40°C). After protease III incubation, the sections were subjected to RNA-scope hybridization assay. The probes were designed and produced by BioTechne depending on targets' gene ID. To start the hybridization, the RNA probe(s) (in our study, a fluid mixture of probes named C1, C2, and C3 channels) was added to the slide with sections. Incubation was going on in a HybEZ™ Oven (Bio-Techne co.) for 2 h at 40°C. After hybridization incubation, the slides were washed using 1 × RNA-scope® Wash Buffer. Then the sections were incubated with RNA-scope® Multiplex FL v2 Amp 1, 2, and 3 (for 30 min/30 min/15 min, respectively) sequentially at 40°C to amplify the signal. For signal development, RNAscope® Multiplex FL v2 HRP-C1, HRP-C2 and HRP-C3 were added to the sections sequentially (incubation time 15 min) in our RNA-scope® Multiplex study. For revealing signals, TSA-diluted Opal™ 520, 570, and 690 fluorophores were added to sections after HRP-C1, C2, and C3, incubating the sections for 30 min each at 40°C. When the three Opal fluorophores were assigned to each channel, in our experiment, three channels C1, C2, and C3 were assigned to Na/K-ATP1A1, Na/K-ATP1B1, and Na/K-ATP1B3 or Cx probes (Ref: 567981 RNAscope probe Hs-GJD2, Ref: 541391 RNAscope probe Hs-GJB6, Ref: 539891 RNAscope probe Hs-ATPase alpha 1, Ref: 568261 RNAscope probe Hs-ATPase Beta 1) (Table 3). After each fluorophore incubation and rinse with 1 × RNA-scope® Wash Buffer, RNA-scope® Multiplex FL v2 HRP blocker was added and incubated in oven for 15 min at 40°C. Finally the sections were counterstained with DAPI and the slides cover-slipped with ProLong® Glass Antifade Mountant (Thermo fisher Scientific). RNA-scope ISH produces puncta of signal that represent a single mRNA transcript (Grabinski et al., 2015).

**Table 3:** RNA probes used in the present investigation.

**Gene**	**Species**	**Gene ID**	**Chromosome location**	**Cat#**	**Company**
GJD2	Human (h)	57369	15q14	567981	BioTechne (b)
GJB6	h	10804	13q12.11	541391	b
Na/K-ATPase β1	h	481	1q24.2	568261	b
Na/K-ATPase β3	h	483	3q23	568271	b
Na/K-ATPase α1	h	476	1p13.1	539891	b

### MicroCT

MicroCT was used to analyze the 3D anatomy of the nerves in the internal acoustic meatus. We used a diffusible iodine-based technique to enhance contrast of soft tissues for diffusible iodine-based contrast-enhanced computed tomography (diceCT). Increased time penetration of Lugol's iodine (aqueous I2KI, 1% I_2_, 2% KI) offers possibilities to visualize between and within soft tissue structures (Camilieri-Asch et al., 2020). The temporal bone was fixed in a modified Karnovsky's fixative solution of 2.5% glutaraldehyde, 1% paraformaldehyde, 4% sucrose, and 1% dimethyl sulfoxide in 0.13 M of Sorensen's phosphate buffer. Soft tissue contrast was achieved by staining the sample for 14 days as described by Culling (Culling, 1974). X-ray microCT was conducted using a Versa 520 XRM (Zeiss, Pleasanton, CA, USA) running Scout and Scan software (v11.1.5707.17179). Scans were conducted at a voltage of 80 kV and 87 μA, using the LE4 filter under 0.4 × optical magnification and a camera binning of 2. Source and detector positions were adjusted to deliver an isotropic voxel size of 23 μm. A total of 2,501 projections were collected over 360°, each with an exposure time of 1 s. Raw projection data were reconstructed using XM Reconstructor software (v10.7.3679.13921; Zeiss) following a standard center shift and beam hardening (0.1) correction. The standard 0.7 kernel size recon filter setting was also used (Culling, 1974). Images were imported into the 3D Slicer program (Slicer 4.6; www.slicer.org), an open-source software platform for medical image informatics, image processing, and 3D visualization. Images were resized at a scale of 4:1, and opacity and gray scale values were adjusted during volume rendering. The technique allows reconstruction in three dimensions, and bones were made transparent and cropped.

### Transmission and Scanning Electron Microscopy (TEM and SEM)

Four archival specimens collected during surgery were analyzed in Uppsala and Innsbruck; the technique used was previously described (Tylstedt and Rask-Andersen, 2001). Briefly, specimens were fixed in 3% phosphate-buffered glutaraldehyde, pH 7.4, and rinsed in 0.1 M cacodylate buffer, and then immersed in 1% osmium tetroxide at 4°C for 4 h. The specimens were dehydrated and infiltrated with Epon resin in a vacuum chamber for 4 h. Sections were viewed in a JEOL 100 SX electron microscope in Uppsala. For SEM, specimens were placed in 3% sodium phosphate buffered glutaraldehyde and perfused through the oval and round windows. Specimens were coated with a 10- to 15-nm layer of gold-palladium in a BALTECH® MED 020 Coating System and observed with a ZEISS® DSM 982 Gemini Field Emission Electron Microscope operating at 4 to 5 kV (Rask-Andersen et al., 2012).

## Results

**Figure 1:**
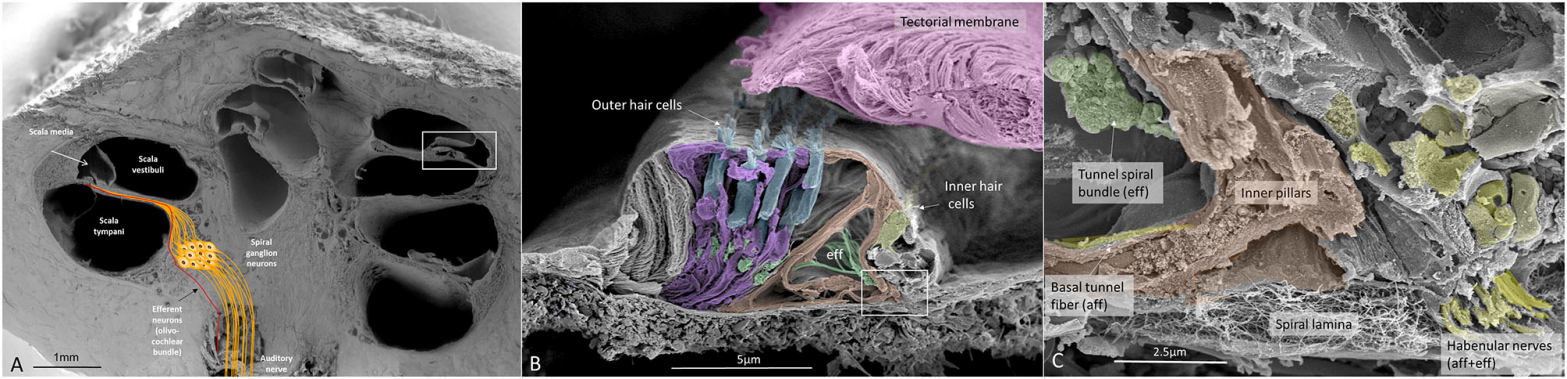
**(A)** SEM of a decalcified hemi-sectioned human cochlea. Framed area shows the OC. The SG (yellow) contains two types of afferent neurons: one innervating outer (5%) and one IHCs (95%). In total, there are about 30,000 nerve fibers (NFs). Efferent NFs (red) also reach and interact with SG cell bodies, IHC nerve terminals, and outer hair cells OHCs. Printed with permission from Hearing, Balance, and Communication 2020, https://doi.org/10.1080/21695717.2020.1807259. **(B)** SEM of a human OC. There are four rows of OHCs and one row of IHCs. Efferent NFs are colored green. **(C)** Higher magnification of framed area in **(B)**. A basal afferent tunnel fiber runs in the inner pillar cell foot. It is an afferent fiber innervating OHCs. IHC afferent terminals are swollen. A similar, but not identical, image was earlier published in Anatomical Record 2012 (Rask-Andersen et al., 2012).

SEM of a hemi-sectioned human cochlea and organ of Corti (OC) is shown in Figure 1. Higher magnification of the organ of Corti shows the multicellular acoustic crest with sensory hair cells and surrounding supporting cells (Figure 1B) and innervation pathway (Figure 1C).

**Figure 2:**
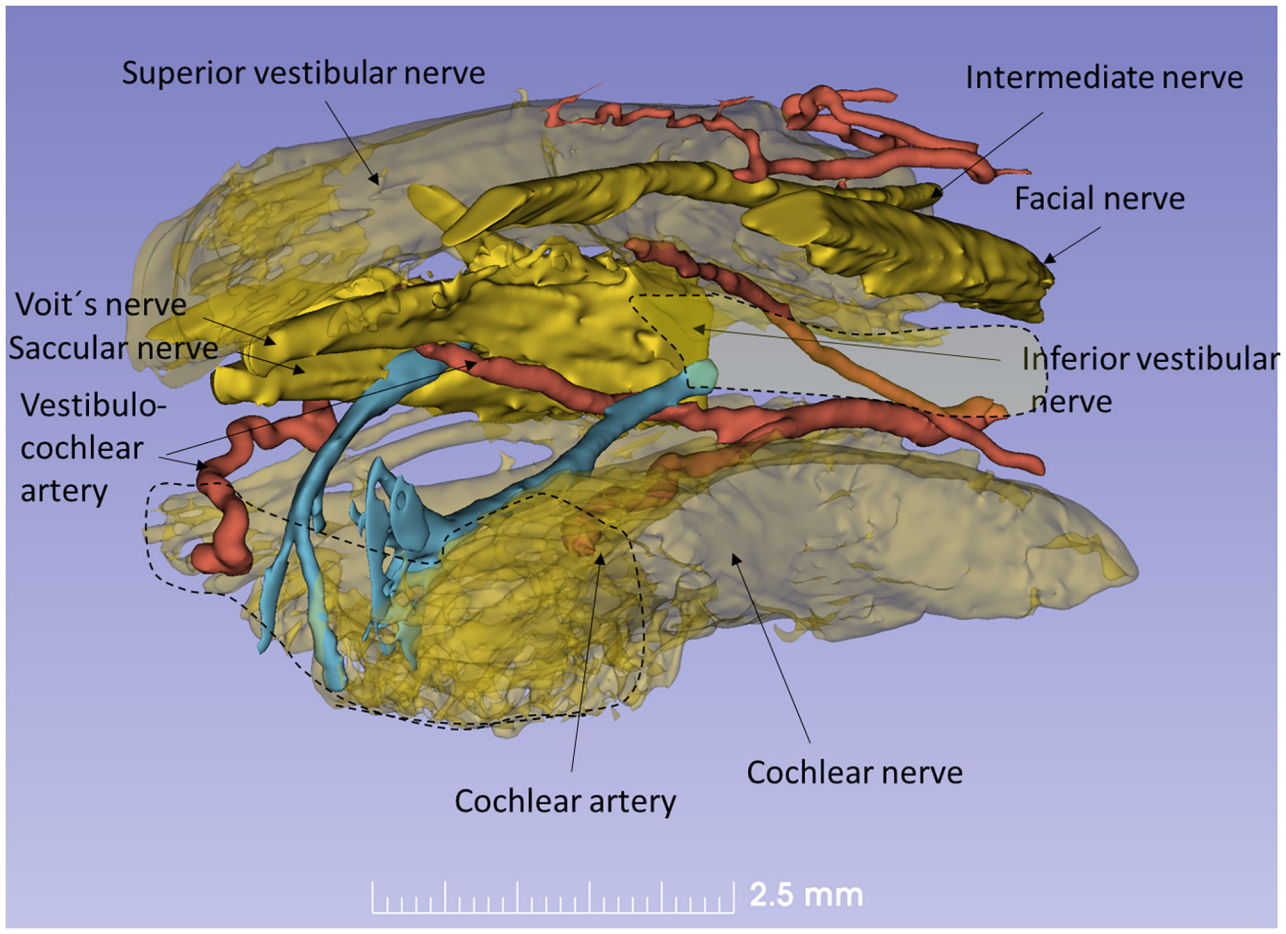
Human efferent innervation. MicroCT, 3D reconstruction and modeling of soft tissue in a right human IAC (anterior-medial view, broken line represents cochlear nerve at fundus). For clarity, some nerves are semi-transparent. An efferent cochlear nerve supply is mediated via the vestibular-cochlear anastomosis of Oort (blue). NFs exit from the inferior vestibular and saccular nerves and reach the cochlea and SG ~3–4 mm from its basal end. Their role in signal modulation, protection, and spatial hearing is still unclear.

**Figure 3:**
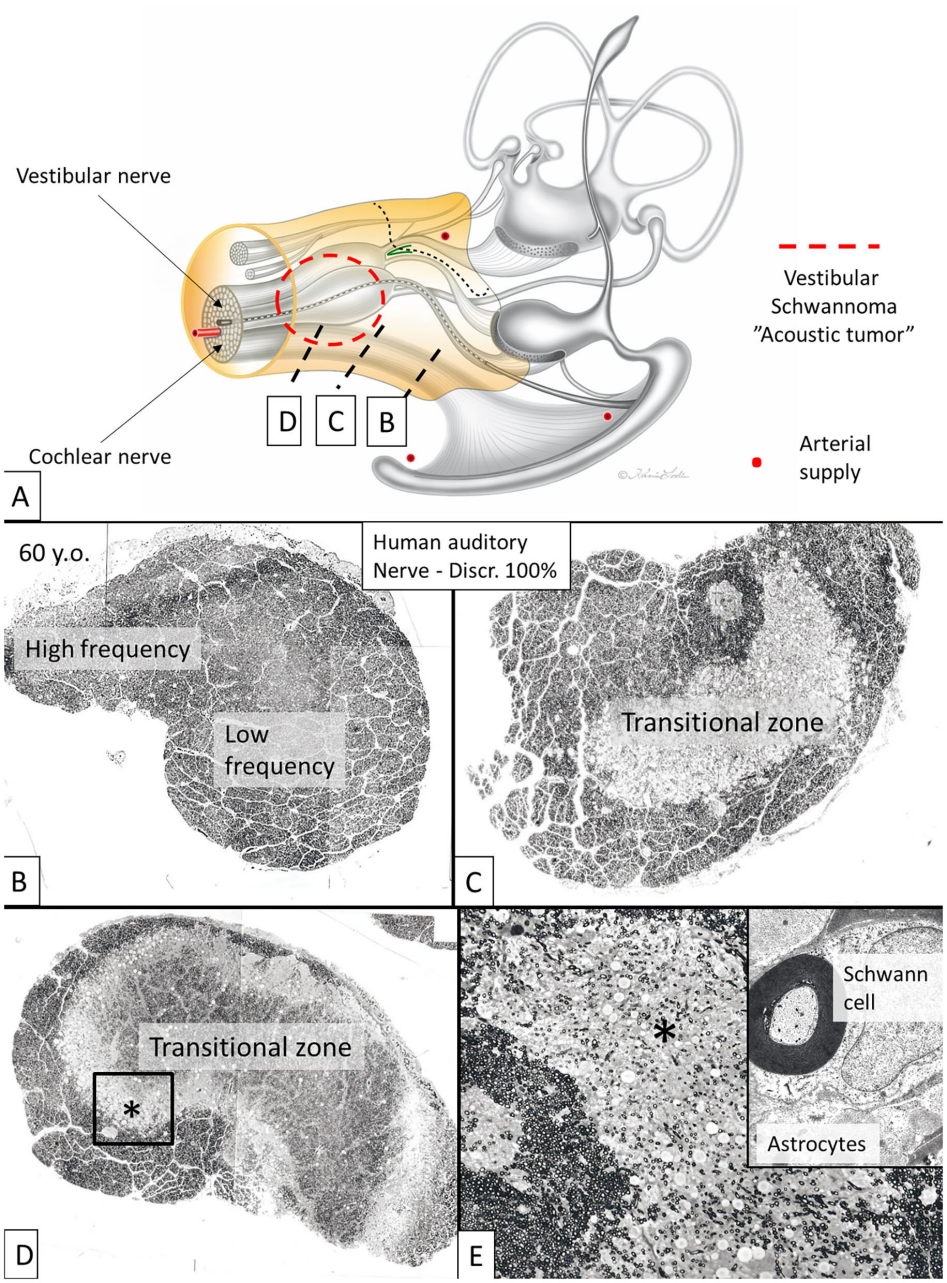
Cross-sectioned human auditory nerve at different levels shown in **(A)**. **(B)** The nerve near the fundus. **(C,D)** A transitional zone with central glia and astrocyte tissue project into the nerve (lucent part). It is surrounded by the peripheral component containing Schwann cells. **(E)** Higher magnification of framed area shown in **(D)**. Inset shows TEM image at the transitional zone with astrocyte tissue (asterisk). Tissue was fixed immediately in 3% buffered glutaraldehyde and post-stained in 1% osmium tetroxide.

**Figure 4:**
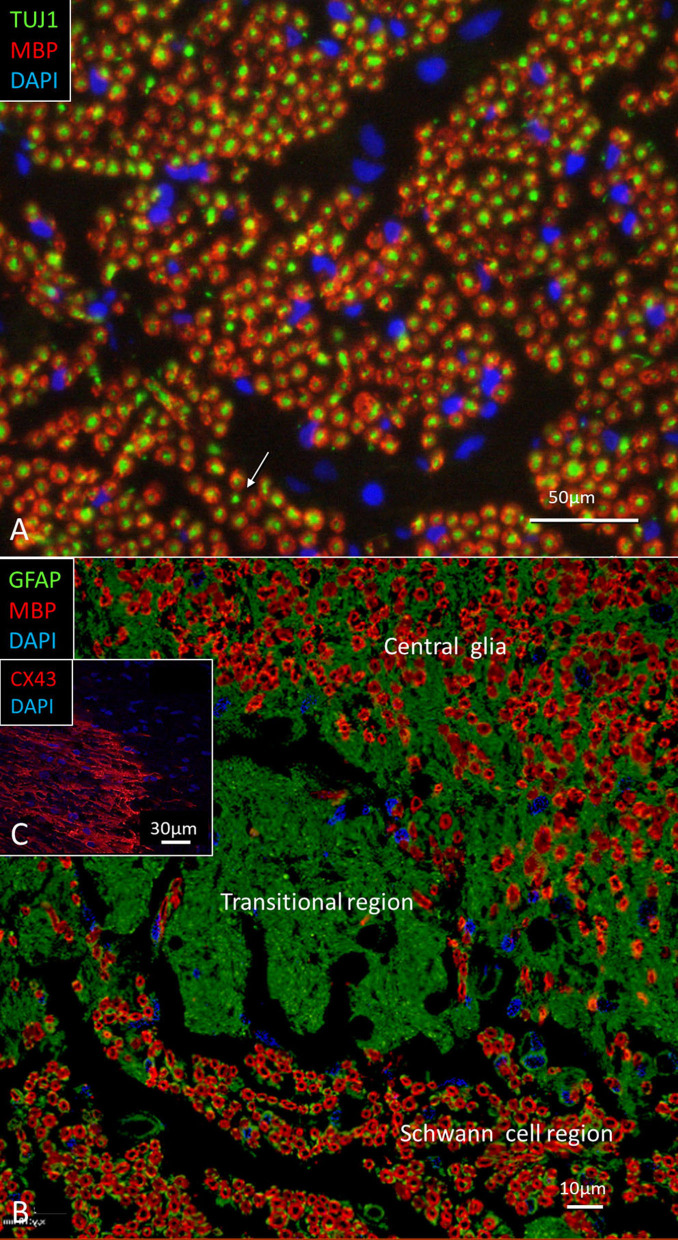
**(A)** Immune staining of a cross-sectioned human auditory nerve corresponding to the level shown in Figure 3B. **(A)** Nerve fibers express the myelin marker MBP and neuron marker TUJ1. Few fibers are unmyelinated (arrow) and may represent NRs. **(B)** Cochlear nerve at the transitional zone (corresponding to level shown in **C**). Astrocytes stain positive for GFAP (green) and Cx43 (inset). MBP, myelin basic protein; TUJ1, tubulin-1; GFAP, glia-fibrillary acidic protein; Cx43, connexin43.

The nerve and vascular supply to the human hearing organ is demonstrated using microCT. It reproduced both the afferent and efferent nerve supply within the internal acoustic meatus. 3D modeling demonstrates the vestibular-cochlear anastomosis of Oort and blood vessels in a right ear in Figure 2. Several efferent bundles leave the inferior vestibular nerve to reach the cochlea 3–4 mm from its basal end. At surgery it was also possible to remove and directly fix a human cochlear nerve for LM and TEM as well as for immunohistochemistry (Figures 3B–E, 4). Cross-sections at different levels show the nerve both near the fundus and at the transitional zone after glutaraldehyde fixation and osmium staining. The transitional zone contained a central lucent part with glia and astrocyte tissue projecting peripherally into the nerve. It was surrounded by a part with Schwann cells (Figures 3C–E). Immune staining of a cross-sectioned human auditory nerve near the fundus is shown in Figure 4A and shows that nerve fibers express the myelin marker MBP and neuron marker TUJ1. Only a few single fibers were unmyelinated and are believed to represent NRs. Though, peripherin antibody staining was not performed so it cannot be excluded entirely that they represent type II afferent fibers originating from the small ganglion cells passing to the brain. At the transitional zone astrocytes stained positive for GFAP and Cx43 (Figure 4B and inset). Surprisingly, a few Nav1.6-positive ganglion cells were occasionally found in the distal part of the IAC along nerve fascicles (not shown here). Their axonal initial segments (AISs) express Nav1.6.

### The Spiral Ganglion and Expression of Nav, Kv, Caspr1, and Ankyrin G

**Figure 5:**
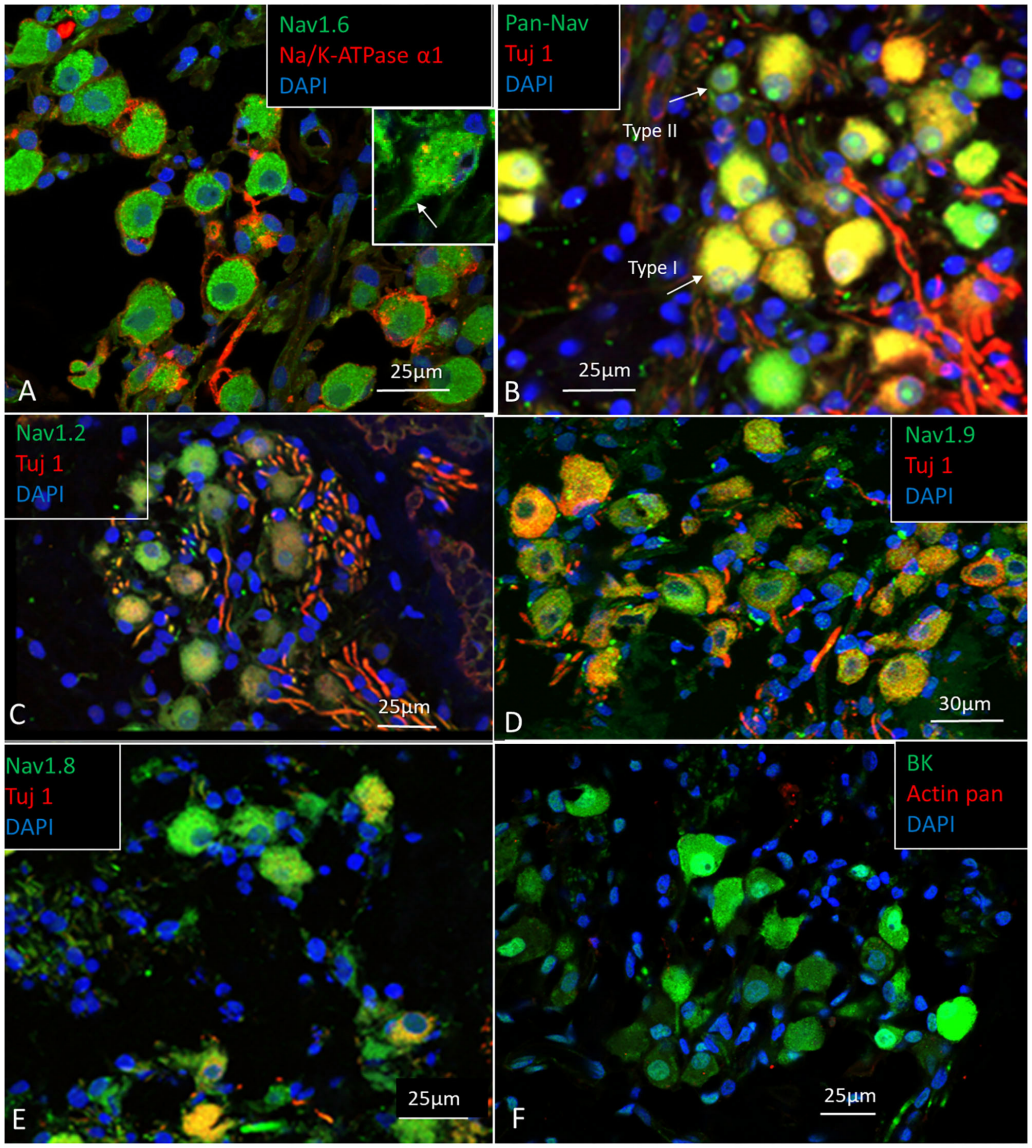
**(A–E)** Expression of Nav, and TUJ1 in the human SG. The large cell bodies show various expression of Nav channels, mostly restricted to the cell bodies and the AIS. **(F)** Spiral ganglion cell bodies also expressed calcium-activated potassium channel (BK channel). Fixation in 4% PFA.

The SG is located in a 13–14 mm long bony canal in the modiolus called Rosenthal's canal (RC) (Ariyasu et al., 1989; Stakhovskaya et al., 2007; Li et al., 2018). It is well-defined in the basal turn only. It contains afferent large ganglion cells (LGCs) or type I cells (87–97%) innervating the IHCs and small ganglion cells (SGCs) or type II cells (3–13%) that innervate the outer hair cells (OHCs) (Arnold et al., 1980; Arnold, 1987; Spoendlin and Schrott, 1989; Rosbe et al., 1996). Large or type I spiral ganglion cell soma are surrounded by non-myelinating satellite glial cells (SGCs) and lack expression of MBP. In the apex, SGCs form a more or less complete honeycomb layer. SGCs were surrounded by a basal lamina expressing lamininβ2 and collagen IV and were connected by gap junctions (GJs) expressing Cx43. Expression of voltage-gated sodium channels is summarized in Table 4. Large and small spiral ganglion cell bodies expressed Pan-Nav, Nav1.6, and TUJ1 with no particular concentration in the plasmalemma (Figures 5A,B). Large ganglion cell bodies also expressed Nav1.2, 1.7, 1.8, and 1.9 but were not present in NRs (Figures 5C–E). The intensity of Nav staining varied among cell bodies. There was no expression of Nav1.1 and 1.3. Type I spiral ganglion cell bodies expressed calcium-activated potassium channels (BK-channel) (Figure 5F).

**Table 4:** Immunohistochemical expression of Nav channels.

**Channel type /Location**	**Nav1.1**	**Nav1.2**	**Nav1.3**	**Nav1.6**	**Nav1.7**	**Nav1.8**	**Nav1.9**
Hair cells	N/A	N/A	N/A	+_(OHCs)_	N/A	N/A	N/A
SG cell body	–	+	–	+	+	+	+
Dendrites	–	+	–	–	–	–	–
Axons	–	+	–	–	–	–	–

*N/A; not analyzed. +; positive expression. –; no expression*.

**Figure 6:**
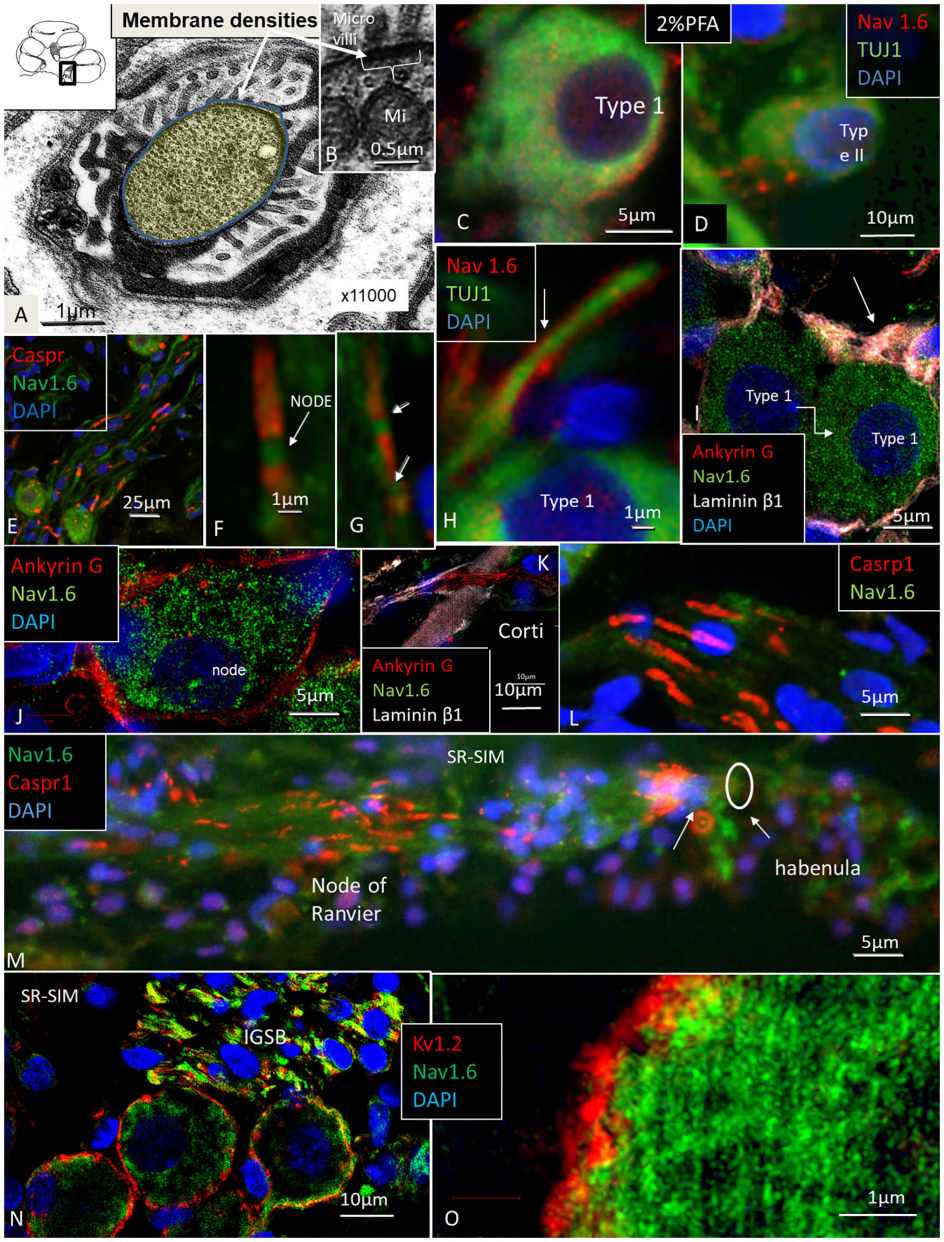
**(A,B)** TEM of a cross-sectioned node/paranode in the basal RC. Axoplasm is stained yellow. Radially oriented arrays of Schwann cell microvilli contact the axolemma **(B)**. Mi, mitochondria. **(C)** Co-expression of Nav1.6 and TUJ1 in a large type I cell after fixation in 2% PFA. **(D)** Small type II cell with adjoining Nav1.6-positive fibers fixed in 2% PFA. **(E,F)** Nav1.6 expression in NRs. **(G)** A double NR (arrows). **(H)** Type I AIS (arrow) expresses Nav1.6. **(I,J)** Ankyrin G expression in Type I cell axon hillock (arrow) and plasmalemma. **(K)** Ankyrin G expressed in neurons at the HP. **(L)** Caspr1 is expressed in neurons beneath the HP. **(M)** Radial NFs express Caspr1 beneath the HP (arrows) and at NRs. **(N)** Expression of Kv1.2 and Nav1.6 in type I SGNs. Efferent fibers in the IGSB also express Nav1.6. **(O)** Higher magnification shows expression of Kv1.2 in the type I SGN plasmalemma.

Several RN/para-nodes were identified in RC and a cross-sectioned RN can be seen with TEM in Figure 6A. Radially oriented arrays of Schwann cell microvilli can be seen to contact the axolemma (Figure 6B). The microvilli are known to contribute to and maintain Nav channel clustering in NRs (Gatto et al., 2003; Zuo et al., 2008). A thick coat beneath the plasma membrane forms assembly of cytoskeletal proteins. If the PFA concentration was lowered to 2%, Nav1.6 plasmalemma staining increased and in the AIS, but at the same time cell preservation weakened (Figures 6C,D,H). Nerve terminals and varicosities on small ganglion cell bodies expressed Nav1.6 (Figure 6D). The NRs expressed Nav1.6 and was limited by contactin-associated protein 1 (Caspr1) at the paranodal region (Figures 6E–G). “Double” NRs were noted in the RC (Figures 6F,G). Ankyrin G was expressed around the LGC bodies (Figures 6I,J). Including a fourth channel showed that Ankyrin G co-expressed with the basal lamina protein lamininβ2. The basal lamina was often crumpled at axon hillock regions where both laminin β2 and ankyrin G were expressed. Ankyrin G was also expressed in NFs at the habenula perforata (HP) (Figure 6K). HP also strongly expressed Caspr1 beneath the basilar membrane (Figures 6L,M). Several first NRs were found beneath the basilar membrane that expressed Caspr1 while staining of Nav1.6 was generally faint. Unmyelinated efferent nerve fibers belonging to the intra-ganglionic spiral bundle (IGSB) also expressed Kv1.2 and Nav1.6 (Figure 6N). Kv7.1 (KCNQ1) was discretely expressed in the LGCs (not shown), while Kv1.2 labeled their plasmalemma (Figure 6O). If the ganglion cell bodies expressed also Kv1.1 could not be settled with certainty.

### Immunohistochemistry and TEM of the Spiral Lamina and Habenular Canal

**Figure 7:**
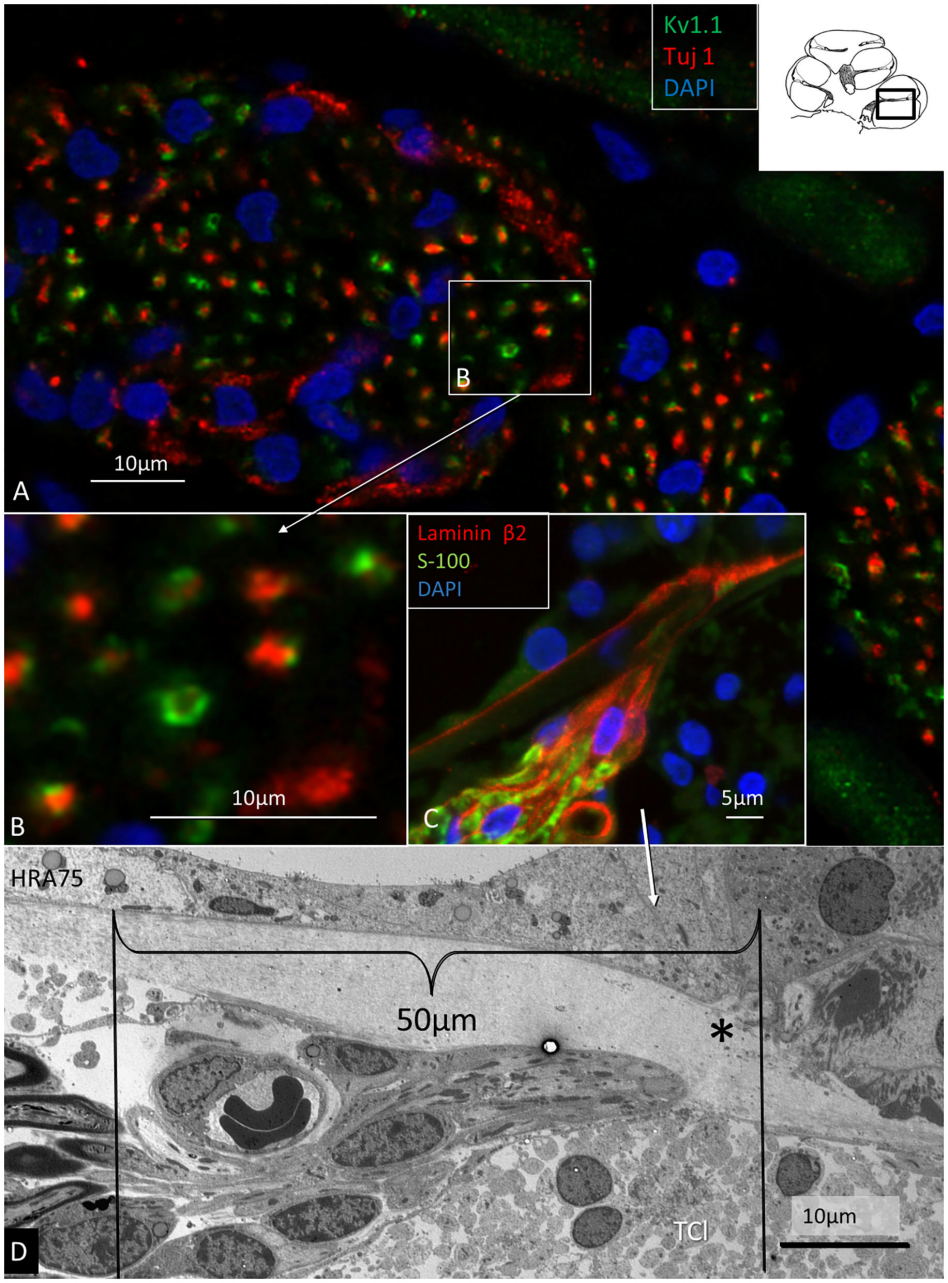
Immunohistochemistry **(A–C)** and TEM **(D)** of spiral lamina NFs in a human cochlea. **(A)** Double-labeling of TUJ1 and Kv1.1. Framed area is magnified in **(B)**. **(C)** Radial section at the HP with a fiber bundle expressing lamininβ2 and S-100. **(D)** TEM of radial NFs beneath the HP (asterisk). The length of the unmyelinated region is around 50 μm. The unmyelinated NFs are rich in mitochondria. A blood capillary is located typically near the nerve bundle. The basal lamina is folded at the habenular opening. TCL, tympanic covering layer.

In the spiral lamina fibers, the NRs and juxta-para-nodes expressed Kv1.1 margined by Caspr1 as can be seen in Figures 7A,B. The radial myelinated afferent fibers were Nav1.6-negative, except at the NR. Their fiber diameter was around 2 μm. The spiral lamina also contained groups of very thin myelinated and unmyelinated fibers running spirally. They strongly expressed TUJ1 and Nav1.6. These neurons are thought to represent efferent fibers and were earlier shown to be synaptophysin-positive (Khalifa et al., 2003). They also enter the OC through the foramina nervosa. Single radial unmyelinated fibers can also be seen to run in the spiral lamina using SEM (not shown here). They have a diameter of less than 0.5μm. Whether they express Na1.6 could not be established with certainty. Immunohistochemistry of the spiral lamina beneath the HP is shown in Figure 7C. At this region the afferent NFs lose myelin and coalesce into bundles embedded in S-100 positive glial cells that follow the fibers through the canal. It could not be established with certainty if the NFs beneath the HP expressed Kv1.1 and Kv1.2. Radial sectioning with TEM showed the afferent NFs beneath the HP which were rich on mitochondria and surrounded by glial cells and a thin basal lamina expressing laminin β2 (Figures 7C,D). The lamina tapered the inner wall of the habenular canal. The length of the canal was 10–15 microns. The length of the unmyelinated region was ~20–30 μm with fibers having a diameter around 1μm. The fibers were rich in mitochondria, and a blood capillary was typically situated where the nerves enter the canal. In the canal, the neurite diameter diminished to around 0.5 μm (Figure 7D). The diameter of the habenular canal varied and was around 6 × 4 microns (area 20–40 μm^2^). NFs almost completely filled the canal and were surrounded by a thin glial sheet into the OC. Type II afferents and efferents could not be separated in the habenular canal.

### Expression of Na/K-ATPase in the Human Auditory Nerve

**Figure 8:**
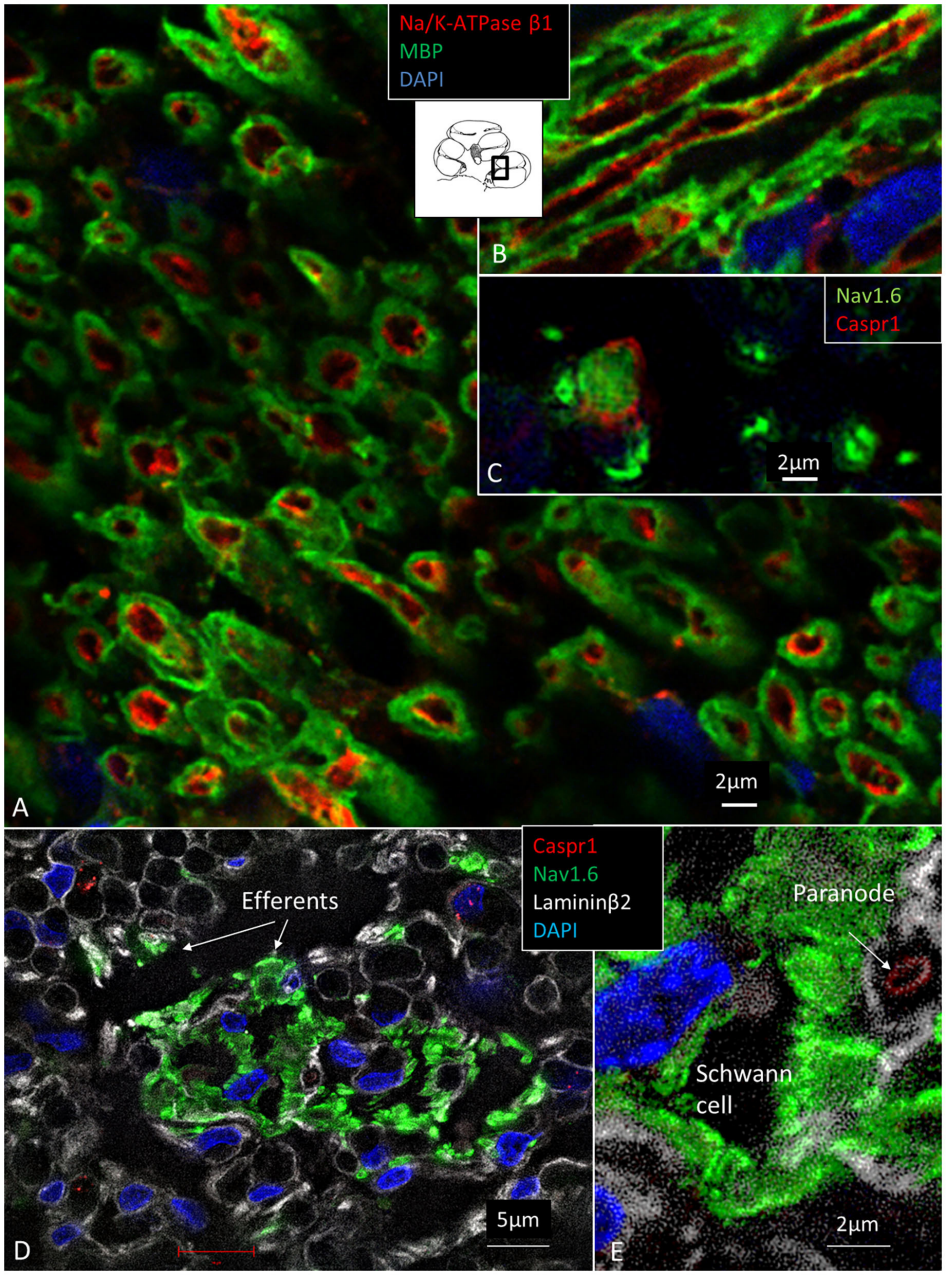
**(A)** Spiral lamina NFs cross-sectioned and double-labeled with antibodies against MBP and Na/K-ATPase β1. **(B)** Longitudinal section. Axolemma expresses Na/K-ATPase β1 all the way. **(C)** Cross-sectioned axons labeled with antibodies against Nav1.6 and Caspr1 at and near a NR. **(D)** The spiral lamina contains bundles of thin NFs expressing Nav1.6 believed to represent efferent nerve fibers. The larger myelinated NFs are Nav1.6-negative. **(E)** Higher magnification of Nav1.6-positive NFs shown in **(D)**. A fourth channel (white) shows lamininβ2 expression in the basal lamina. MBP, myelin basic protein; Nav1.6, voltage-gated sodium channel 1.6; Caspr1, contactin-associated protein 1.

The expression of Na/K-ATPase in the human cochlea was recently presented in a separate study (Liu et al., 2019). Table 5 summarizes the expression of various isoforms in the human cochlea. Na/K-ATPase β1 subunit was heavily expressed generally in the human cochlea, mostly combined with the α1 isoform. Neurons, however, expressed the β1 subunit combined with α3, while SGCs expressed the α1 isoform. LGC plasmalemma strongly expressed Na/K-ATPase α3/β1. The central and peripheral myelinated axons stained positive for Na/K-ATPase α3/β1 isoforms all the way (Figures 8A–C). The groups of small spirally running myelinated and unmyelinated lamina fibers strongly expressed Na/K-ATPase α3/β1 (not shown) and also Nav1.6 (Figures 8D,E).

**Table 5:** Immunohistochemical expression of Na/K-ATPase isoforms.

	**ATPase α1**	**ATPase β1**	**ATPase α2**	**ATPase β2**	**ATPase α3**	**ATPase β3**
**SGN-p (Type I)**	**-**	**+(RNA-scope +)**	**-**	**-**	**+**	**N/A (RNA-scope +)**
SGCs	+	–	–	?	–	N/A
Axons	–	+	–	–	+	N/A
Dendrites	–	+	–	–	+	N/A
Nerve endings	–	+	–	–	+	N/A
ISB	–	+	–	–	+	N/A
OSB	–	+	–	–	+	N/A
TCF	–	+	–	–	+	N/A
TBF	–	+	–	–	+	N/A
SGN (Type II)	–	?	–	–	+	N/A

*SGN-p, Large type I spiral ganglion neuron perikarya; SGC, Satellite glial cells; Axons and Dendrites are large SGN's neurites; Nerve endings, SGN terminal fibers, and nerve endings inside OC are unmyelinated. OSB; outer spiral bundle. ISB; inner spiral bundle. TCF; tunnel crossing fibers (efferents). TBF; tunnel basal fibers; afferents Type II fibers. N/A: not analyzed. +; positive expression. –; no expression*.

**Figure 9:**
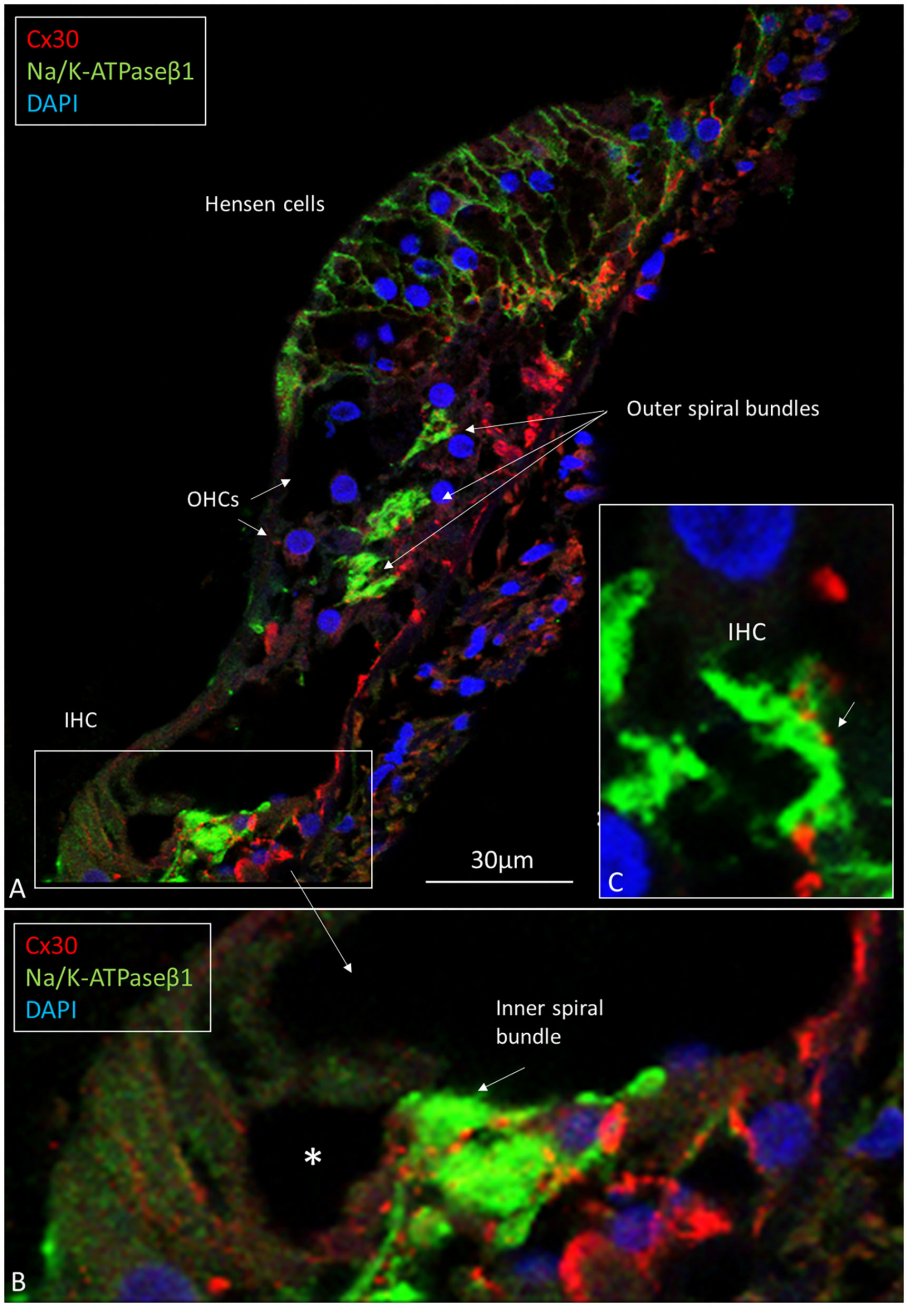
**(A)** CM of the human OC double-labeled with antibodies against Na/K-ATPase β1 (green) and Cx30 (red). Inner and outer spiral bundles express Na/K-ATPase β1 with mostly separate Cx30 puncta **(B,C)**. *IHC nerve terminals are swollen.

In the organ of Corti, both afferent and efferent nerve terminals strongly expressed Na/K-ATPase α3/β1 (Figure 9). Nav1.6 co-expressed with Na/K-ATPase β1 in inner, outer, tunnel spiral bundles, and tunnel crossing fibers. The highest activity of Na/K-ATPase β1 in the OC was at the IHC/nerve junction, inner and outer spiral bundles, Hensen cells, marginal cells, type II fibrocytes and spiral prominence. RNA-scope hybridization confirmed gene transcripts of Na/K-ATPase ATP1B1 and even ATP1B3 in LGC bodies. The ATP1B1 was confined to the cell periphery, while ATP1B3 transcripts were distributed more evenly in the cytoplasm and cell nuclei. The localization of ATP1B1 and ATP1B3 encoding Na/K-ATPaseβ1 and β3 in human large, type I SG cell bodies are seen in **Figure 12**. ATPB1 gene expression is concentrated near the cell membrane while ATP1B3 is mostly expressed in the cell nuclei. SR-SIM shows intense expression of the Na/K-ATPaseβ1 in the plasmalemma of large ganglion cells lying closely together. SR-SIM verified both genes encoding β1 and β3 Na/K-ATPase isoforms in the same cell.

### TEM of Human Organ of Corti

**Figure 10:**
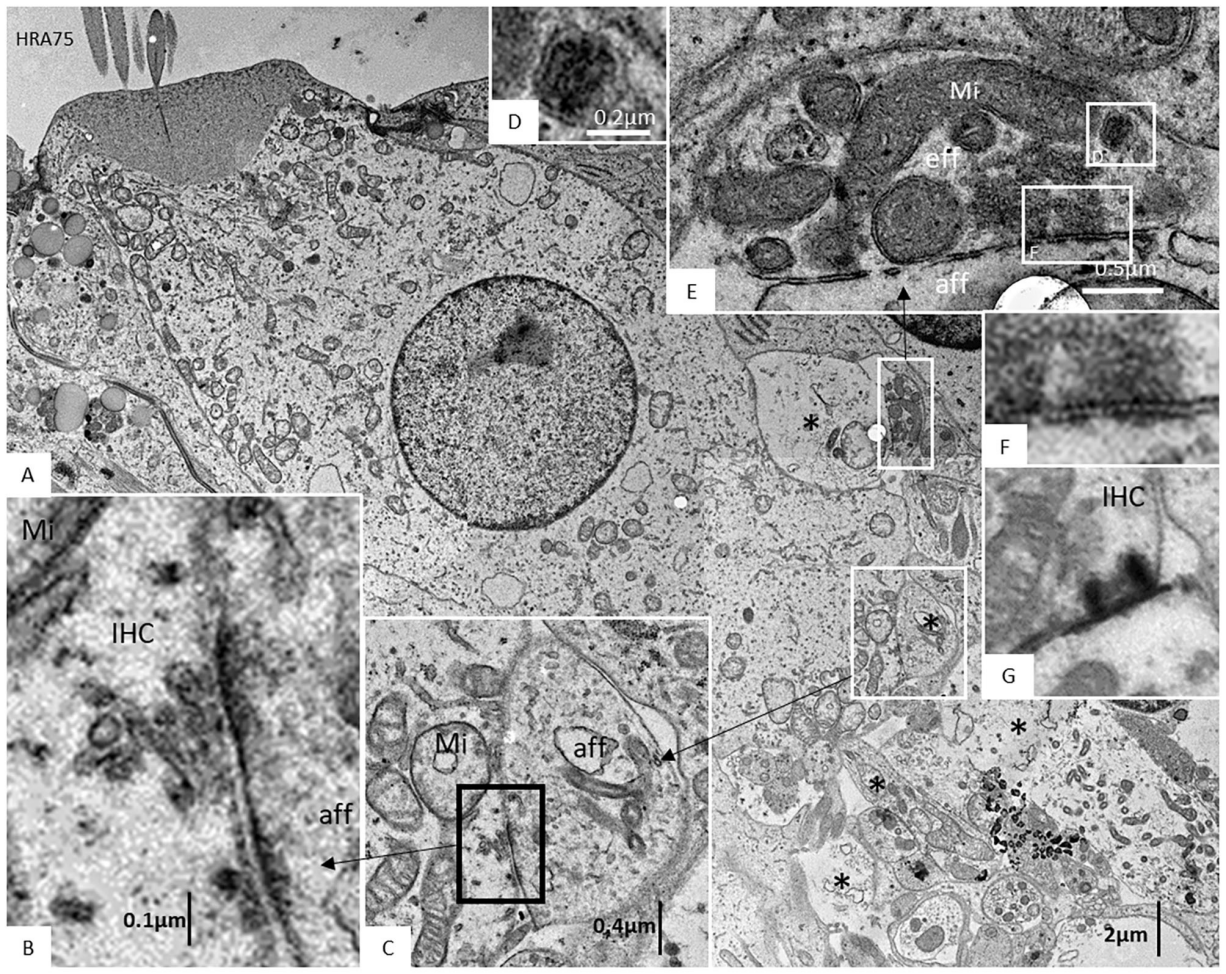
**(A)** TEM image of a human IHC. A myriad of afferent (*) and efferent nerve terminals are located at the basal pole. **(B,C)** Afferent boutons (aff) have different morphology with multiple synaptic plaques. There are large numbers of mitochondria (Mi) in the IHC synaptic region. **(D–F)** An efferent axo-synaptic contact is shown with multiple synaptic vesicles, mitochondria, and large dense-core vesicles **(D)**. Some terminals face more than one ribbon synapse **(G)**. Fixation was done directly in the surgical room in oxygenated 3% buffered glutaraldehyde and then post-stained in 1% osmium tetroxide.

The basal lamina accompanied neurites for a short distance inside the OC, with “entrance gate.” Thereafter, the basal lamina turned back and followed the basal region of the organ. Neurites contained several mitochondria, while surrounding glial cells showed electron-dense bodies, rER, and glycogen granules. Each nerve fiber entered the organ of Corti through minor openings in the surrounding glial cell layer. Ribbon synapses occurred in both IHCs and OHCs, and not infrequently, several ribbons were found against the same nerve terminal in both IHCs and OHCs. TEM images of a well-preserved human IHC with numerous afferent and efferent nerve terminals located at the basal pole are shown in Figure 10. Afferent boutons are shown to have different morphology with multiple synaptic plaques. Typically is the large numbers of mitochondria in the basal cytoplasm of the IHC synaptic region. Efferent axo-synaptic contact show multiple synaptic vesicles and large dense-core vesicles. A systematic study of the ultrastructure of the IHC receptor-neural junction is under way.

### TEM and Connexin30 in Human Spiral Ganglion Cells

**Figure 11:**
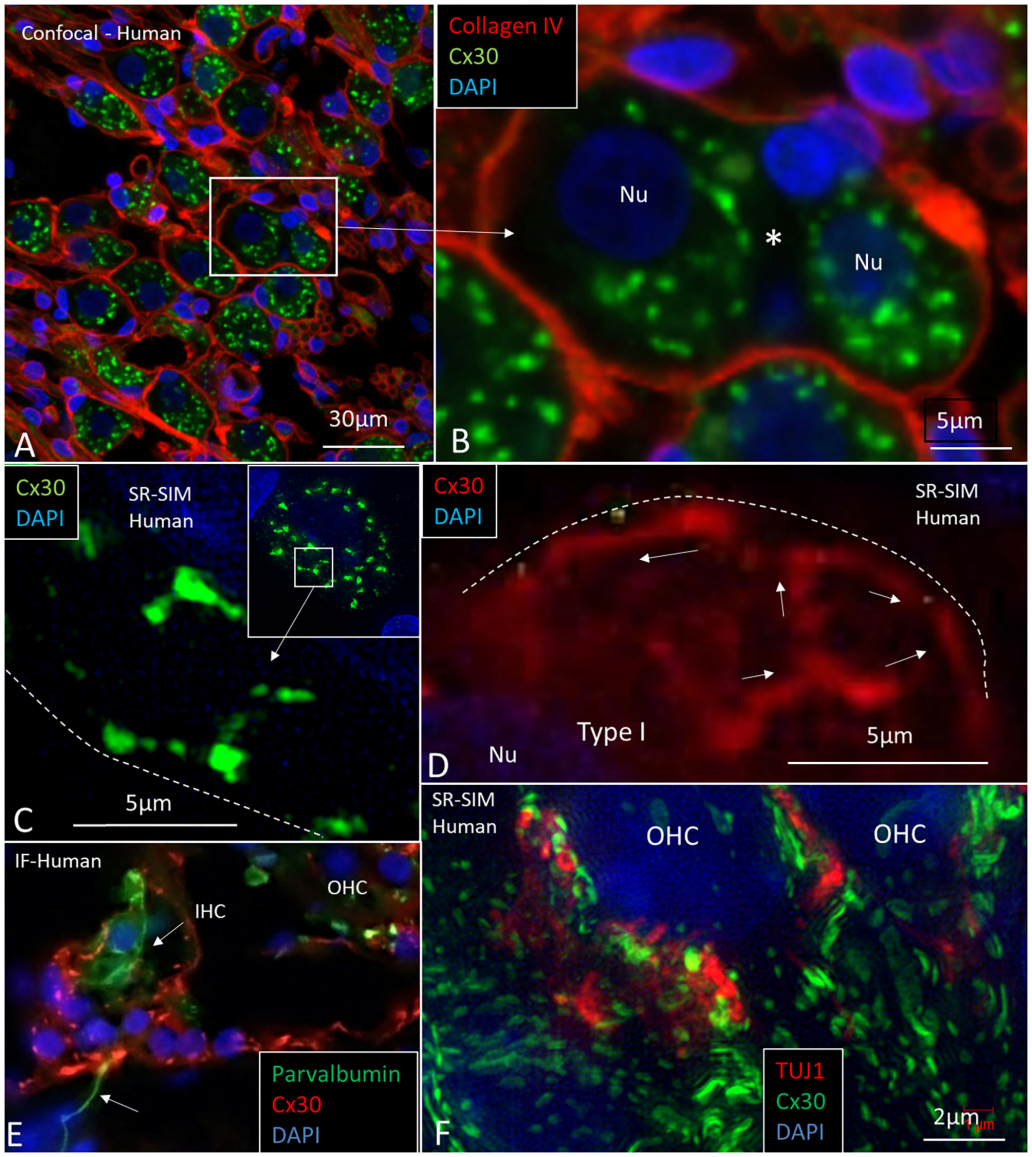
CM and SR-SIM of human SG and OC. **(A)** Collagen IV and Cx30 co-staining shows a basal lamina “honeycomb” layer in the apical portion of the cochlea richly expressing Cx30. Framed area is magnified in **(B)**. **(B)** A SGC with no basal lamina is seen between the two large SG cell bodies (*). **(C,D)** SR-SIM shows labeled Cx30 near the plasmalemma (broken lines). **(E)** Inner hair cell with afferent (arrow) shows expression of parvalbumin but no Cx30. Surrounding cells heavily express Cx30. **(F)** SR-SIM at lower poles of OHCs. Neurons express TUJ1 but there is no co-expression with Cx30. Nu: nucleus.

**Figure 12:**
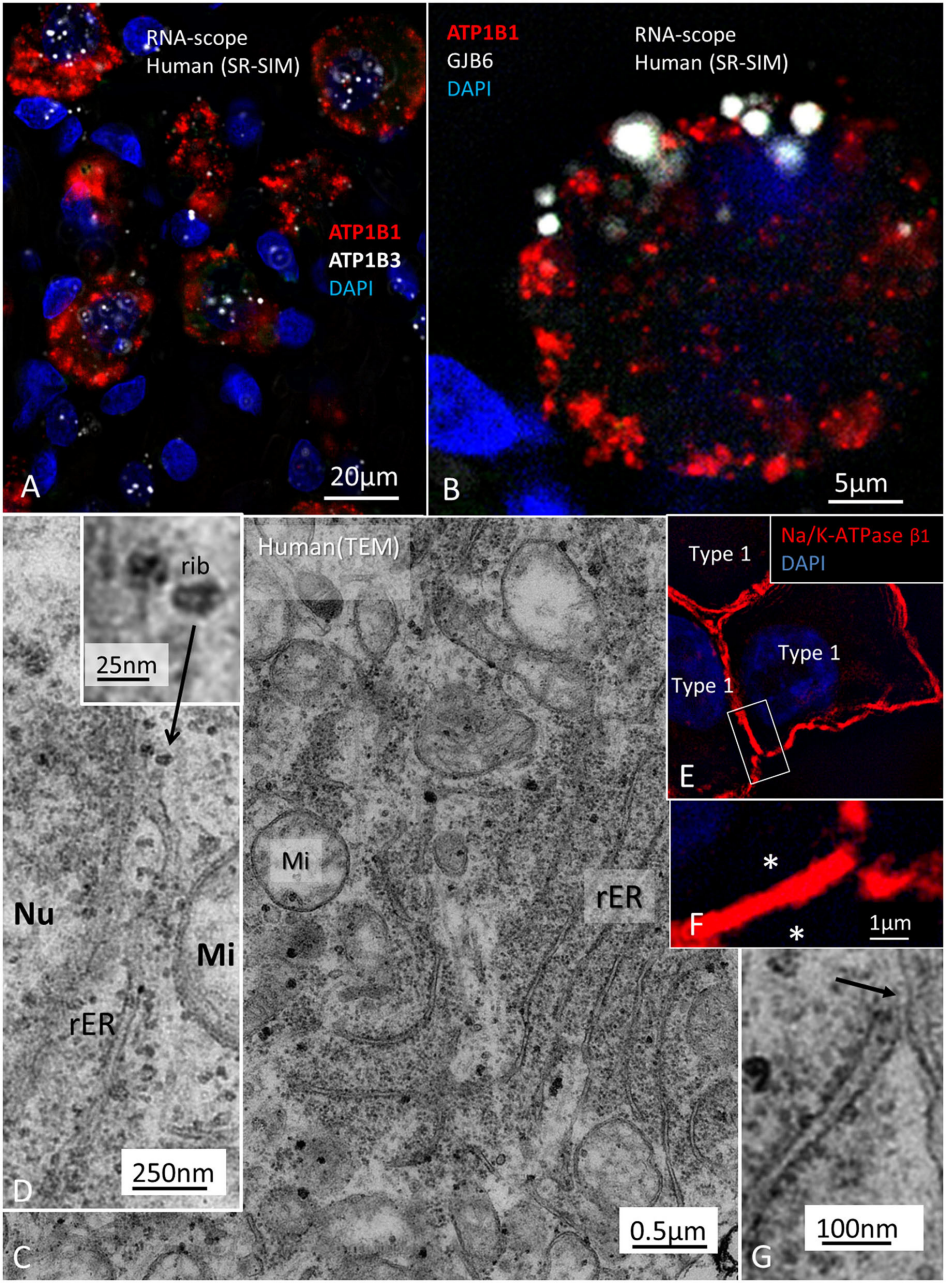
**(A)** Localization of ATP1B1 and ATP1B3 in human large, type I SG cell bodies encoding Na/K-ATPaseβ1 and β3. ATPB1 gene expression is most concentrated at the periphery near the cell membrane while ATP1B3 is mostly expressed in the cell nuclei. **(B)** GJB6 encoding Cx30 protein is expressed near the plasmalemma of a type I ganglion cell. **(C)** TEM image of a type I ganglion cell shows many mitochondria (Mi) and extensive rough endoplasmic reticulum (rER) and free ribosomes in the cytoplasm. **(D)** Several ribosomes are located at the nuclear envelope. **(D)** shows ribosome (rib) in higher magnification. **(E)** A rER is closely associated with the plasmalemma (arrow). **(F)** SR-SIM shows expression of the Na/K-ATPaseβ1 protein in the plasmalemma of three large ganglion cells. The cell membranes lie close to each other (asterisks). Framed area is magnified in **(G)**. Nu; cell nucleus.

Large ganglion cell bodies surprisingly expressed Cx30. Cell bodies had a “mulberry-like” appearance at immunofluorescence (Figures 11A–D). Laser CM and SR-SIM with 3D reconstructions demonstrated both neural markers TUJ1 and Cx30. An elaborate network of Cx30 protein extended between the nuclear envelope and cell periphery (Figures 11C,D). A rich network of rough endoplasmic reticulum (rER) was observed with TEM (Figures 12C,D,G). RNA-scope hybridization confirmed gene transcripts of GJB6 in LGC bodies (Figure 12B). Cx36 could not be verified with RNA-ISH. Human IHC and OHCs and neurons heavily expressed parvalbumin but there was no co-expression with Cx30 (Figure 11E). Likewise, SR-SIM demonstrated no co-expression of Cx30 and TUJ1 in nerve elements beneath the OHCs (Figure 11F). Cx30 could be demonstrated in the OC, spiral limbus, and lateral cochlear wall in guinea pigs and pig but not in the SGNs (Supplementary Figure 1, Supplementary Video 1). The rich expression of Cx26 and Cx30 in the human OC is demonstrated in Supplementary Figure 2A and Supplementary Video 2.

## Discussion

This study presents some information on the anatomy of the human auditory nerve including its molecular constituents. Results suggest that initial spike generators are located beneath the IHCs in humans. However, additional mechanisms seem to be essential for the filtering of complex sounds that may be challenged by various pathological conditions. The cochlear nerve relays acoustic information to the brain along homogenously sized myelinated fibers. The retro-cochlear meatal part contains a highly vulnerable transitional zone where synchronized nerve signaling may be compromised by external influences. Efferent nerve fibers from the vestibular nerve reach the cochlear nerve at its entrance near bony perforations of the fundus. Micro-CT results herein and synchrotron phase-contrast imaging (Mei et al., 2020) expose the 3D anatomy of the associated arteries extending from the cranium. Despite the obvious difficulties in studying well preserved human material, these emerging molecular analyses show the specific distribution of VGICs and expression of connecting proteins among physically interacting ganglion cell bodies. It seems to suggest that human acoustic nerve signaling may be partly different from most laboratory animals.

### Human Receptor-Neural Segment—An Intriguing Spike Generator

The innervation pattern points to the IHC system is the main transfer of acoustic information to the CNS, while the OHCs provide hair cell-based amplification to increase auditory sensitivity and frequency selectivity (Rhode, 1971; Kemp, 1979; Flock et al., 1986). A detailed examination of the human receptor-neural complex is challenging due to its extraordinary anoxia-sensitivity and nerve terminal swelling. Therefore, studies of transduction channels and excitatory activity in human sensory cells are challenging. The human cochlea contains 3,400 IHC receptors that relay acoustic information to the brain via 30,000 nerve fibers (Retzius, 1884; Guild et al., 1931; Wright et al., 1987). Graded transduction currents and voltage-gated Ca^2+^ channels activate a sublime system of multi-vesicular ribbon synapses releasing hundreds of quantized transmitter vesicles per second to glutamate/AMPA-receptors in each nerve terminal with remarkable endurance (Moser and Beutner, 2000; Glowatzki and Fuchs, 2002; Grant et al., 2010). Modulated synchronized release produces excitatory postsynaptic potentials (EPSPs) (Geisler, 1981; Siegel and Dallos, 1986; Moser and Beutner, 2000; Nouvian et al., 2006; Safieddine et al., 2012), and APs are generated to transfer sound features as phasic, fast adapting signals with extraordinary temporal and spectral resolution (Siegel, 1992; Fuchs, 2005; Rutherford et al., 2012). Remarkably, human IHC afferent boutons were associated with more than one synaptic ribbon, contrary to most laboratory animals where each fiber seems to make only one contact with the IHC (Nadol, 1988; Kantardzhieva et al., 2013). In humans, one terminal can make multiple synaptic contacts with a single IHC or two adjacent IHCs (Nadol, 1983). Bodian (1978) though, found dual ribbon synapses in non-human primates (Bodian, 1978). In turtles and frogs, hair cell synapses have been extensively studied, and it was found that many ribbon synapses converge on a single afferent, but each nerve fiber forms several synaptic terminals onto one to three hair cells (Keen and Hudspeth, 2006), and no synapses were associated with more than one synaptic thickening (Schnee et al., 2005). Presynaptic ribbons are also present in retinal photoreceptors where they exhibit sustained release of neurotransmitter activity that reaches several postsynaptic targets, such as horizontal cells and bipolar neurons at some distances (Matthews and Fuchs, 2010). Our results may suggest that signal transmission could be more “multifaceted” in humans and non-human primates, and whole-mount immunohistochemistry and SR-SIM can add new information about principal signaling and aberrations (Viana et al., 2015; Liu et al., 2019).

**Figure 13:**
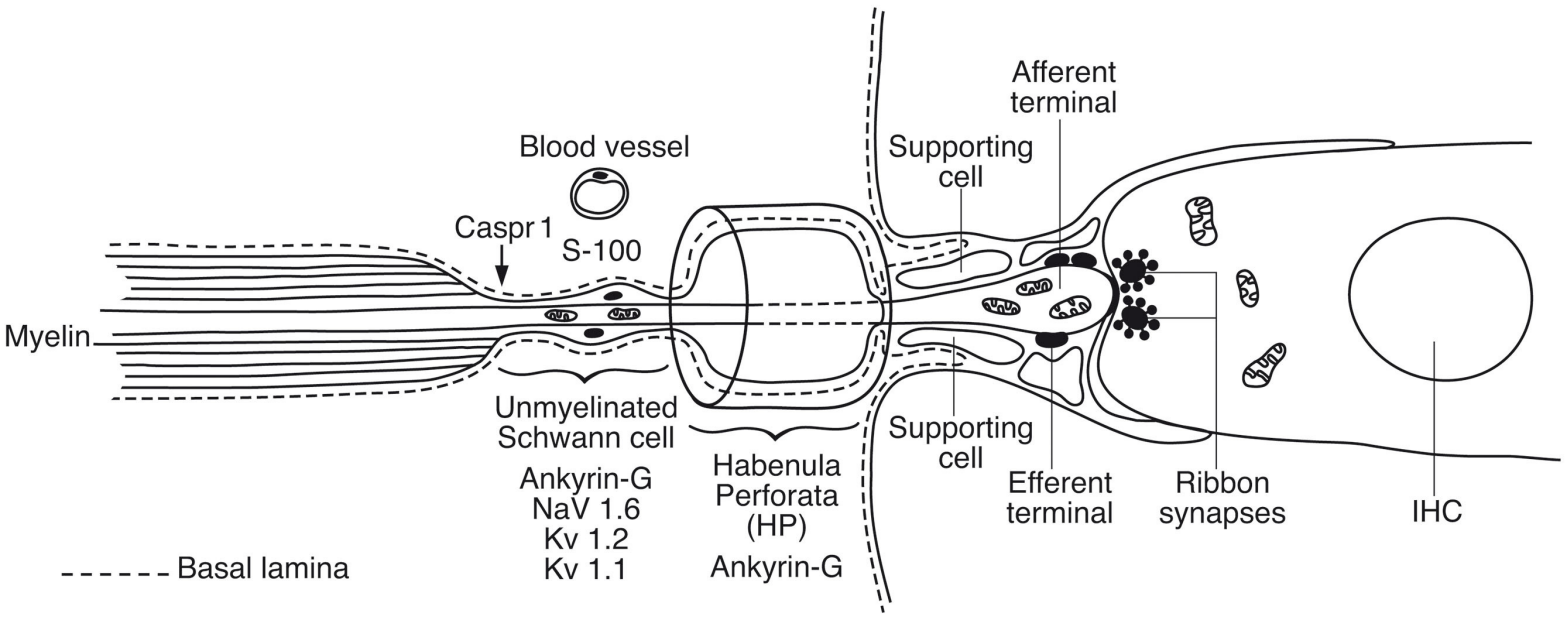
Graphic illustration of the principal organization of the human inner hair cell receptor-neural junction. A basal lamina margins the neural pathway that coalesces with the organ of Corti. Action potentials are believed to be generated in the sub-receptor zone beneath the habenular canal. In rodents Nav1.6 is localized in a hemi-node consistent with the location of spike generation (Hossain et al., 2005). There are also low- and high-voltage activated potassium channels essential for adaptation and regulation of AP activity (Smith et al., 2015; Kim and Rutherford, 2016).

In human, we found a diversity of nerve terminals and neurites beneath the IHC. Afferent terminals and efferent fibers heavily expressed Na/K-ATPase α3/β1, which is essential for repolarization after spike activation. Different sized afferents may represent those with variable thresholds and spontaneous rates (Nadol, 1983; Merchan-Perez and Liberman, 1996). Postsynaptic excitatory currents are known to arise within the OC (Grant et al., 2010) and may generate APs beneath the HP. Changes in the molecular machinery of ribbon synapses may lead to impaired speech perception, such as cochlear neuropathy (Roux et al., 2006; Safieddine et al., 2012). Injury caused by age or noise may result in “hidden hearing loss,” (Schaette and McAlpine, 2011; Kujawa and Liberman, 2015) although conclusive evidence for noise-induced cochlear synaptopathy in humans remains elusive (Bramhall et al., 2019). Animal work may show that spontaneous rate and sensory coding of the type I afferents depends on the size of ribbon synapses and Ca-channels density (Sheets et al., 2017). Further studies of the human ribbon synapses and diversity of nerve terminals are needed along the cochlear spiral and are underway at our laboratory. The organization of the human inner hair cell receptor-neural junction based on results obtained so far is given in Figure 13, Supplementary Figure 3.

Somatic sensory signaling is known to create receptor potentials and firing at hemi-nodes (Bewick and Banks, 2014; Carrasco et al., 2017) that open sodium channels to produce APs in the first NR (Loewenstein and Ishiko, 1960). Control of firing often occurs before myelination (Bender and Trussell, 2012). Each human IHC is innervated by ~10–15 afferent nerve fibers (Nadol, 1988) that pass through the foramina nervosa along a 34 mm long spiral. The present data point to the sub-habenular region as hemi-node and spike generator in humans. A limitation was that Nav1.6 could not be unequivocally established beneath the IHCs, but the scaffold proteins Caspr1 and Ankyrin G were identified. Ankyrin G-binding motifs are important for sodium channel clustering and targeting of Nav1.6, Kv7.2, and Kv7.3 as well as Na/K-ATPase and Na/Ca^2+^-exchanger (JoséGarrido et al., 2003; Pan et al., 2006). The reason for the lack of detection of Nav1.6 in this region is not known at this stage. In the rat, this region clearly expresses Nav1.6 and low- and high-threshold-voltage-gated potassium channels as well as Ankyrin G and Caspr1 (Lacas-Gervais et al., 2004; Smith et al., 2015; Kim and Rutherford, 2016). According to Kim and Rutherford Kv1.1 was present ubiquitously in axons and somas in the mature rat and enriched at juxta-para-nodes, Kv2.2 was expressed in internodes, Kv3.1 only in hemi-nodes and nodes and Kv7.2 and 7.3 in myelinated and unmyelinated segments in the osseous spiral lamina and beneath the IHCs. Nav1.6 was found to co-localize with Kv3.1b at hemi-nodes and nodes and Nav1.1 was located in hemi-nodes only (Kim and Rutherford, 2016). Hence, the visualization of VGIC may be influenced by the pattern of myelination and further analyses in man seem required. Hossain et al. showed that Nav1.6 channels in mice are located in afferent axons central to the HP and in unmyelinated afferents and terminals in the OC (Hossain et al., 2005). OHC afferents also expressed Nav1.6 channels. The spike generator was thought to reside near the postsynaptic bouton before axons myelinate. The unmyelinated efferent axons and endings on the inner and outer hair cells expressed Nav1.2 but never in the type II afferents running on the floor of the tunnel or in the outer spiral bundle or endings (Hossain et al., 2005). In human, the unmyelinated NFs beneath the HP displayed a large number of mitochondria constantly associated with a distinct vascular supply. It suggests that this is a metabolic “hot-spot” that could be consistent with its involvement in the generation of action potentials.

### Can the Human Auditory Nerve Also Fire Through Electric Synapses?

The present findings raise queries as to whether IHC afferent activity can be modulated by mixed electric and chemical synapses. If so, cell coupling may play a role in short delay depolarization and fast signal conduction. Only a few GJ channels (which are morphologically undetectable) can drastically adjust electric transmission acting independent of the resting membrane potential (Bennett, 2009). Signaling through electric synapses is 10 times faster than chemical synapses (synaptic delay 0.2 ms), and Cx36 is the principal neuronal connexin in the mammalian CNS (Bennett, 2009). However, the gene transcript GJD1 could not be detected in our study. At double-labeling, Cx30 was not co-expressed with the TUJ1 or parvalbumin marker in the human OC. Double-labeling with Cx30 and Na/K-ATPaseβ1, however demonstrated Cx30 to be widely, but separately, expressed beneath OHCs and IHCs (Figure 9). Furthermore, there was no co-expression of parvalbumin and Cx30 or TUJ1 and CX30 in neurons beneath IHCs and OHCs (Figures 11E,F). The results give no evidence that electric synapses exist in the human organ of Corti.

### Human SG—A Spike Generator and Acoustic Filter?

The human SG differs distinctly from other vertebrates, suggesting that electric activity is fundamentally different. Large or type I cell soma are unmyelinated and surrounded by SGCs (Kimura et al., 1979, 1980; Ota and Kimura, 1980; Spoendlin and Schrott, 1988; Tylstedt et al., 1997; Liu W, et al., 2014). These neurons terminate at the IHCs, while the small unmyelinated type II neurons innervate the OHCs (Spoendlin, 1972). Some authors have even suggested that the small neurons represent cholinergic parasympathetic fibers (Ross, 1969; Ross and Burkel, 1973). A third type was described in humans by Rosbe (Rosbe et al., 1996).

The percentage of compact myelination in large type I cells in different vertebrates is 85–100% (goldfish, rat, guinea pig, rabbit, and monkey), while in humans only 2–6% are myelinated, and mostly in older individuals (Arnold, 1987). The Ly5.1 mouse strain is the only rodent model reported to have “human-like spiral ganglion neurons” and may be useful for studying synchronous nerve activity (Jyothi et al., 2010). Myelination may secure a fast unbroken nerve conduction across the ganglion to the CNS. Signal speed may be slowed down, but the unmyelinated cell soma and pre- and post-soma segments expressing Nav1.6 may serve as additional spike generators modulated by voltage-gated potassium channels (Kv1.2) (Boulet et al., 2016). In humans, the proximal AISs are unmyelinated, often mitochondria-rich. In mice and other laboratory animals, impedance of the large cell body is thought to be compensated by the pattern of myelination of the cell bodies (Hossain et al., 2005). Hossain et al. (2005) found Nav1.6 expressed at the NRs flanked by Caspr at the para-nodal axoglial junctions, while the cell bodies lacked Nav immunoreactivity. Fryatt et al. used reverse transcription polymerase chain reaction (RT-PCR) and immunohistochemistry to study the distribution of Nav channels in rodent SG neurons (Fryatt et al., 2009). Nav1.1, Nav1.6, and Nav1.7 subunits were expressed in rat ganglion cells, and Nav1.1 and Nav1.6 were expressed in axonal processes suggesting that AIS plays a role in the extension of afferent signals across the SG cell soma. There was no difference in labeling between cell membrane and cytoplasm using RT-PCR. More Nav1.6 and Nav1.7 expressions were found in type I than in type II neurons. There was no expression of mRNA for Nav1.2, Nav1.3, Nav1.8 and Nav1.9 in the rat SGN. In a subsequent study Fryatt et al. showed modulation of VGSCs after noise and mild hearing loss with decreased Nav1.1 and Nav1.6 mRNA expression while Nav1.7 mRNA expression increased by ~20% when compared to control rats (Fryatt et al., 2011).

In the present study, ganglion cell bodies expressed Nav1.2, Nav1.6, Nav1.7, Nav1.8, and Nav1.9, suggesting considerable molecular diversity. Though, the pattern of staining of sodium channels seemed to be highly influenced by aldehyde concentration and cell preservation. The unmyelinated AIS also expressed Nav1.6, and “double nodes” of Ranvier were observed in the RC, suggesting additional modulation of saltatory conduction. Ankyrin G was expressed with laminin β2 and Kv1.2, indicating that electric impulses may be modulated with local voltage amplification to reach threshold (Bender and Trussell, 2012). Ankyrin G is known to gather cell adhesion molecules at the NR and AIS (Kordeli et al., 1995; Dzhashiashvili et al., 2007; Leterrier et al., 2017) and provide means for axon polarity and directional propagation (Rasband, 2010; Leterrier, 2016). Genetic aberrations can cause neuropathy and neural fatigue with enlarged ABR latency and fragmented Kv staining (Lacas-Gervais et al., 2004). Smith et al. found heteromeric Kv1.2 and Kv1.1 channels co-expressed in neurons that may control initiation and propagation of APs in the cochlea as well as Kv3.1b subunits in pre- and post-somatic NRs (Smith et al., 2015). We were not able to localize Kv3.1 or to establish if Kv1.1 and 1.2 were co-expressed.

**Figure 14:**
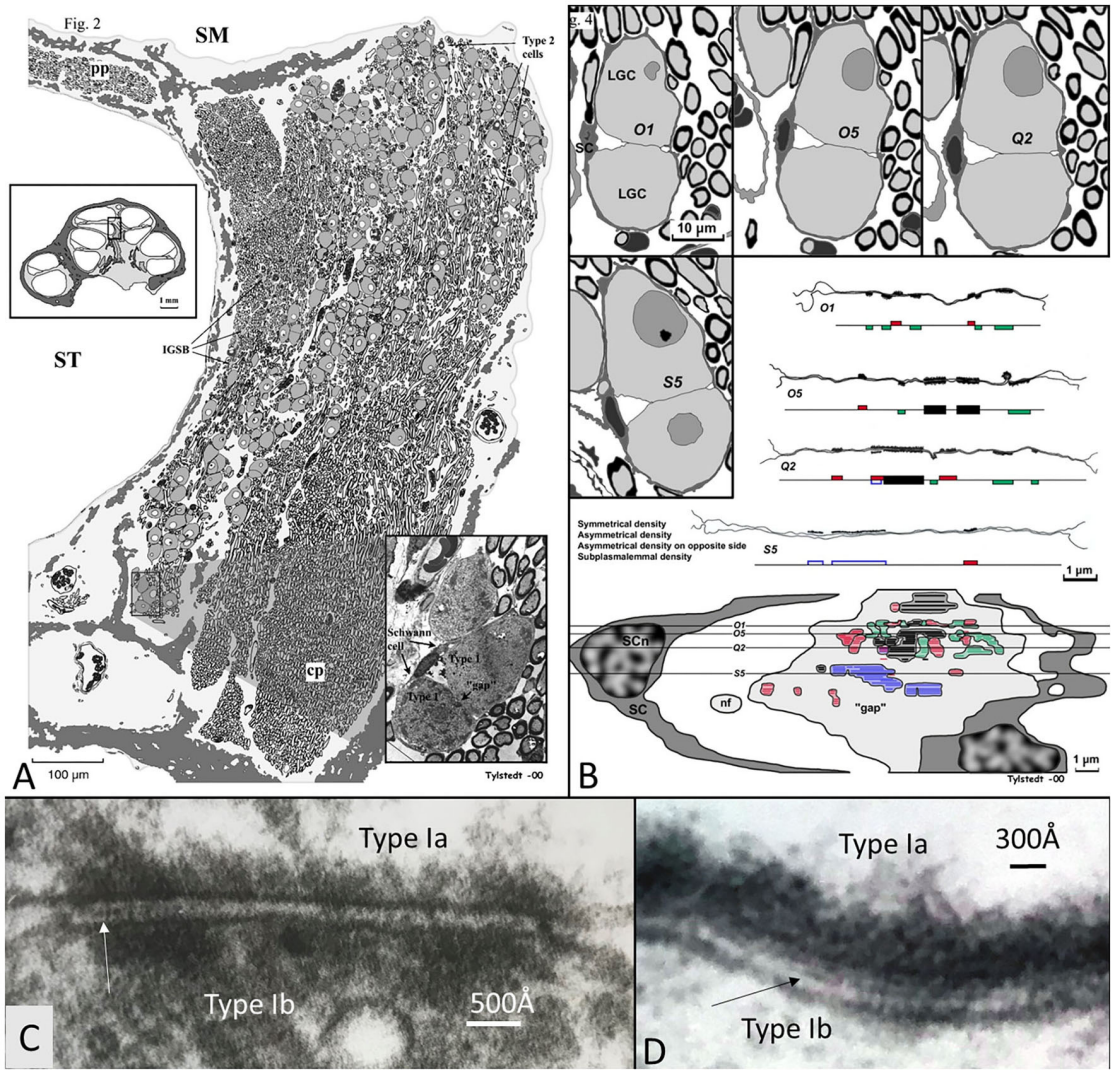
**(A)** 3D model of membrane specializations between two human type I SG cell bodies in the apical turn of a human cochlea (voice fundamental frequency F_0_ ~ 100–250 Hz, 630–730°, normal subjective hearing). Two type I cells were serially sectioned and graphically reconstructed. Surrounding satellite cells (SCs) show a “gap” in the SC interface between the two cells with several membrane specializations. These are both symmetrical (black) and asymmetrical with different polarity (red and green). Some areas show sub-plasmalemmal densities (blue). **(C,D)** High-power TEM of somato-somal membrane densities shown in **(B)**. Magnification ×160,000. Occasionally, a thin precipitous lamina is seen in the intercellular cleft (arrows). Fixed in oxygenated fluorocarbon containing 2% glutaraldehyde solution and 0.05 M sodium phosphate buffer (Tylstedt and Rask-Andersen, 2001). Published with permission from Kluwer Academic Publishers, License Number 4877550695963.

Nerve fiber synapses were previously observed on the small SG cells in the human cochlea (Kimura et al., 1979; Rask-Andersen et al., 2000). LGC bodies also demonstrate synapse-like membrane specializations (Rask-Andersen et al., 1997; Tylstedt et al., 1997), including unique axo-somatic contacts (Tylstedt et al., 1997) otherwise not found in sensory ganglia (Pannese, 2020). Synaptic vesicles are lacking, but accretion of mitochondria suggests specialized neural interaction. These membrane densities are also present among clustered cell bodies where no separating glia layer exists, as demonstrated in Figure 14. This image shows a graphical 3D reconstruction from serial thin sections of membrane specializations between two human type I SG cell bodies in the apical turn of a human cochlea. Surrounding satellite cells show a “gap” in the interface between the two cells with several membrane specializations. These are both symmetrical and asymmetrical with different polarity. The findings may suggest that cell soma interaction is possible for processing of acoustic information. It may also infer a greater plasticity and complexity of cell signaling.

Similar asymmetric densities at opposing junctional membranes are found at synaptic junctions in OHC afferents in primates devoid of synaptic micro-vesicles (Bodian, 1978) and ribbons in the cat (Dunn and Morest, 1975). In these atypical synapses, transmitter vesicles were thought to play a minor role, and quantal chemical transmission was challenged. As in other sensory ganglia, our findings suggest that GJ proteins may be involved in nerve transmission even if electric synapses or GJ plaques were not identified. Moreover, the Cx30 protein was only verified in humans. RNA-scope demonstrated Cx30 gene transcripts confirming earlier Cx30 antibody labeling in the human LGCs (Liu et al., 2009). It suggests that Cx30 may play a role in inter-neuronal communication, seemingly associated with Na/K-ATPase. SR-SIM surprisingly labeled both the β1 and β3 subunits of Na/K-ATPase in the same cell (Liu et al., 2020) and in *in situ* hybridization RNA-scope also localized both genes. It was proposed that β-subunits may play a role in “gluing” cells together (Geering, 1991), essential for cell clustering. β1 was previously found to be co-expressed with α2 and β2 isoforms in the human brain (Tokhtaeva et al., 2012).

Notably, in a recent publication Luque et al. performed a comparative study of the distribution of the unique voltage-gated hyperpolarization-activated cyclic nucleotide-gated (HCN) channels among mammalian species (Luque et al., 2021). These channels have a reverse voltage-dependence activated by hyperpolarization and may generate “pacemaker currents” in heart muscle cells. They form homo- or hetero-tetramers and various subunits (HCN1-4) exist (Wahl-Schott and Biel, 2009). Besides in the OC, these channels were found in neuron clusters of the human SG suggesting a function of synchronization of timing cues. Particular intense staining of HCN 1, 2, and 4 was noted at adjoining cell membranes which may boost ephaptic coupling, synchronizing AP firing similar to that described earlier in the brain (Han et al., 2018).

### Cochlear Injury and Spiral Ganglion Cell Signaling

In sensory ganglia, primary afferents do not seem to function independently but can depolarize via neighboring neurons, leading to cross-excitation through activity-dependent coupling (Amir and Devor, 1996). A critical role is played by the SGCs that are coupled by GJs (Hanani et al., 2002; Cherkas et al., 2004). GJs are also present in SGCs in the human SG and express Cx43 (Liu W, et al., 2014). Trans-cellular signaling among SGCs could partially explain remodeling and neural rescue and incomplete Wallerian degeneration following hair cell and dendrite loss, which are decisive in cochlear implantation. In dorsal root ganglia, cell injury increases neuronal coupling by upregulation of GJs and Cx43 so that adjacent neurons can activate together. SGCs may even proliferate (Hanani et al., 2002) together with activation of surrounding scavenger microglia also present in the human SG (Liu et al., 2018). This neuron-to-neuron communication was termed “crossed after-discharge” and does not seem to represent ephaptic crosstalk (Devor and Wall, 1990). Communication increases after axotomy and contributes to neuropathic pain, an analog to tinnitus (Kim et al., 2016). Inhibiting GJ-mediated coupling was proposed to be a way to relieve chronic pain. However, how neurons are connected was unclear, but Cx43 hemi-channels were suspected. Patch-clamp recordings, dye coupling, and Cx43CKO suggested that SGCs participate in coupled activation. GJs permit the spread of intercellular calcium waves important for signal transfer and induction of sensory disturbances (Dublin and Hanani, 2007). Thalakoti et al. (2007) verified for the first time neuron–glia signaling via GJs, and Damodaram et al. (2009) showed that GJs composed of Cx26 proteins likely mediate direct dye coupling of neurons and SGCs in the trigeminal ganglion. In this context, our finding of Cx30 in the LGCs is intriguing. According to Amir and Devor (1996) cross-depolarization is excitatory and increases neurons' input resistance and spiking for sub-threshold pulses and changes chemical mediated membrane conductance. Excitation was modulated by afferent spike activity and voltage-dependent. It could be induced by the elevation of extracellular potassium (Utzschneider et al., 1992) or the release of chemical mediators, such as excitatory amino acids, eicosanoids, and nitric oxide from neuron soma (Amir and Devor, 1996), whose receptors are also widely expressed in humans.

### Cochlear Implantation, Voice Fundamentals, and Phase Locking

**Figure 15:**
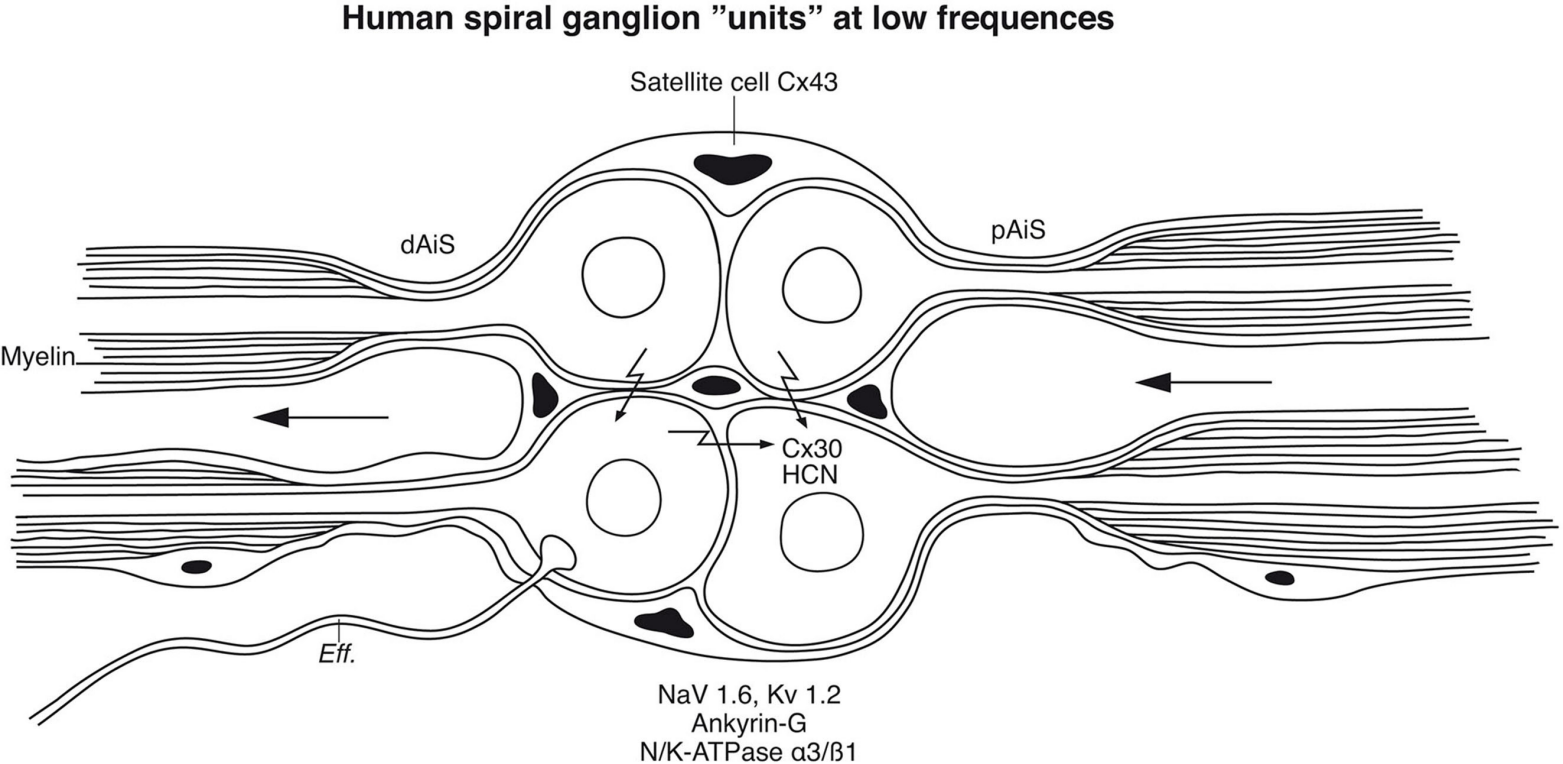
Principal model of large or type I spiral ganglion cell “units” in the human modiolus. Cell bodies and distal (dAIS) and proximal (pAIS) axonal initial segments are unmyelinated in human and surrounded by satellite glial cells. The close proximity between the perikarya may allow inter-cellular communication suggesting an electric filtering at this level. HCN channels were identified in the perikarya with particular intense staining of HCN 1, 2, and 4 at adjoining cell membranes which may boost coupling and synchronize AP firing (Luque et al., 2021). Cx30 was so far not identified in the plasmalemma. Eff., efferent.

Patients with severe sensorineural hearing loss (SNHL) can be treated with CIs to regain substantial hearing and speech comprehension (Michelson, 1971; House and Urban, 1973; Clark et al., 1979; Burian Hochmair-Desoyer and Hochmair, 1981; Hochmair et al., 2015). By placing an electrode array inside the cochlea, plentiful hearing can be regained through electrically generated APs along the NFs. Even congenitally deaf children may achieve speech comprehension and production. This remarkable outcome, despite limited spectral information, suggests that alternate coding principles are involved, such as envelope extraction and temporal cues (Shannon et al., 1995). To improve auditory outcomes with CI, models are created to better understand how external electric stimulation induces neurophysiological responses (Wilhelm-Bade et al., 2009; Bruce et al., 2018). Mapping VGIC and spike generators may reveal new strategies to approach natural hearing (Liu Q, et al., 2014). Today's implants fail to reproduce the traveling wave, spatial resolution from OHCs, and compression of IHC synapse/nerve thresholds through different spontaneous activity. According to Davis and Crozier (2015), ganglion cell bodies are endowed with VGICs that vary along the cochlea, mounting evidence for diverse firing patterns, including thresholds and accommodation. A similar gradient expression may exist in humans. For these variances, potassium channels play an essential role, and in the future, the distribution and molecular diversity along the human cochlear spiral may be examined in more detail. It is notable that Kv1 currents appear only after loosening of the myelin sheath from the axonal membrane, such as the juxta-paranode (Chiu and Ritchie, 1980), a normal situation in human cell soma that could act as a distinguished NR. Un-myelination raises opportunities for wider nerve interaction, cross-excitation, summation, and sub-threshold firing as well as synchronization. In the brain, synchronization depends on spike-frequency adaptations and is essential in auditory perceptual processing (Pantev et al., 1991). In the human ear, several ganglion cell bodies form structural units surrounded by the same SGCs that occasionally allow direct soma-soma interaction. Whether these clusters represent “functional units” representing inputs from one or several IHCs or broader areas is unknown. Acoustic information generated by assemblies of frequency-coded hair cells may be integrated and synchronized to broaden intensity levels and modulate dynamic range. A cell-to-cell communication among similarly tuned cell bodies may coordinate neurite activity and increase tuning sharpness (Figure 15). These units are particularly prevalent in the apical cochlea, where voice fundamentals are coded. A large-scale cross-depolarization may combine place and rate coding for low-frequency temporal excitation, which is essential for speech perception and synchronized phase-locking. This could partially explain why patients lacking peripheral dendrites also discriminate speech through external electric stimulation. Several neurons can fire synchronously (Bennett and Zukin, 2004; Shinozaki et al., 2013) in response to sub-threshold electric activity in clustered neurons (Connors and Long, 2004), such as in the inferior olivary nucleus and inhibitory interneurons of the neocortex, hippocampus, and thalamus through Cx36 (Gibson et al., 1999; Connors and Long, 2004). In the visual system, APs are highly synchronized and mediated by many GJs (Meister et al., 1995) shown to consist of Cx36. More studies are needed to characterize the membrane specializations in the human SG, such as hemi-channel purinergic intercellular signaling, HCN channels, and alternate types of connecting proteins.

Apical neurons are commonly preserved in patients with SNHL, such as in the presbyacusis. However, they are acoustically compressed, obstructing selective stimulation of frequency-coded neurons. A more polarized electric CI stimulation might increase spectral resolution and could even induce activity-based dynamic intercellular communication. This could be induced by the production and modulation of cell connecting protein/molecules establishing novel electrical circuits, a property usually dedicated to the CNS. A broader molecular and genetic diversity of such units may be exposed in future studies, such as those already identified and genetically defined through single-cell RNA sequencing of intensity-coding properties in the murine cochlea (Petitpré et al., 2018; Sun et al., 2018).

## Conclusion

It is challenging to obtain well-fixed human inner ear tissue since it is surrounded by the hardest bone in the body, and neurosensory tissues undergo rapid degeneration. So far, our knowledge of the molecular of the human auditory pathway is fairly limited, and most studies are based on laboratory animals (Kiang, 1980; Rusznák and Szucs, 2009; Davis and Crozier, 2015; Reijntjes and Pyott, 2016; Reijntjes et al., 2019). Results herein indicate that surgically acquired tissue may provide useful information, but it is limited by the relatively small amount of obtainable tissue. It also influences the practical management of control analyses including positive and negative staining and ab-neutralization tests (Burry, 2011). We used RNA scope technique to further validate our findings. A first attempt to use SR-SIM in *in situ* hybridization for gene localization in human cochlear tissue sections was made. Findings suggest surprisingly that molecular expression and nerve signaling may differ in the human auditory nerve compared with that of laboratory animals. A complete understanding of how it relates to various inner ear disorders and strategies for future CI stimulation has not yet been reached. More knowledge about the heterogeneous signal properties in individual neurons, intensity coding, and inter-neural communication and synchrony may be required.

## Data Availability Statement

The datasets presented in this article are not readily available because RNA-scope identified Na/K ATPase genes in the nerve. Requests to access the datasets should be directed to helge.rask-andersen@surgsci.uu.se.

## Ethics Statement

The studies involving human participants were reviewed and approved by the local ethics committee (Etikprövningsnämnden Uppsala, no. 99398, 22/9 1999, cont, 2003, no. C254/4; no. C45/7 2007, Dnr. 2013/190), and patient consent was obtained. The study adhered to the rules of the Declaration of Helsinki. The surgical specimens were from patients suffering from life-threatening posterior cranial fossa meningioma compressing the brain stem. Human cochleas were harvested at major trans-cochlear skull base surgeries, including facial nerve rerouting. The operations were performed at Uppsala University Hospital by a team of neurosurgeons and otoneurosurgeons. Five cochleae were dissected out using diamond drills of various sizes (Table 1). Ethics approval for the microCT project was obtained from the University of Western Australia (UWA, RA/4/1/5210), and the human temporal bones were provided by the Department of Anatomy at UWA. The patients/participants provided their written informed consent to participate in this study. The animal study was reviewed and approved by the local ethics committee (no. C254/4, C209/10).

## Author Contributions

WL performed immunohistochemistry for super-resolution microscopy and performed RNA-scope. AS-F, RG, and ML performed immunohistochemistry of cadaver temporal bones at the Innsbruck University, Austria. GR performed micro-CT of human cadaver. ST supplied (Figure 14) and shared the work related to it. HL performed image processing and 3D visualization of scanned objects provided by SA, HL, and GR. HR-A was the head of laboratory and planned the project, analyzed the images, and wrote the manuscript. All authors contributed to the article and approved the submitted version.

## Conflict of Interest

MED-EL Medical Electronics, R&D, GmbH, and Innsbruck, Austria provided salary support for one research group member (WL) in accordance with the contract agreement with Uppsala University, Sweden during 2018. The funder had no role in study design, data collection and analysis, decision to publish, or preparation of the manuscript. The remaining authors declare that the research was conducted in the absence of any commercial or financial relationships that could be construed as a potential conflict of interest.

## Supplementary Material

Supplementary Figure 1 **(A)** CM of Cx30 expression in the human and guinea pig cochlea. Cx30 is richly expressed in the lateral wall, OC, and spiral limbus in both species. Cx30 is expressed in human SGNs [framed area and **(B)**] but not in the guinea pig SGNs [framed area and **(C)**]. TUJ1; Neuron marker. DAPI; Cell nucleus marker (Supplementary Video 1). Cx30 is expressed in the human SGNs.

Supplementary Figure 2 **(A)** Immune fluorescence and SR-SIM (inset) show expression of Cx26 and Cx30 in the human OC. Cx30 is heavily expressed at the inner and outer hair cell region and among Hensen cells. SR-SIM shows that Cx26 and Cx30 are separately expressed RNAscope and four channel SR-SIM show localization of ATP1A1, ATP1B1 and GJB6 in the OC **(B,C)** and the lateral wall **(D,E)**. Gene transcripts are expressed both in the cytoplasm and cell nuclei (Supplementary Video 2). 3D visualization of the CX26 and Cx30 expression shown in Figure 2A.

Supplementary Figure 3 Illustration of the human habenular canal opening into the organ of Corti based on SEM and TEM. Neurons are accompanied by thin glial cells from which they exit surrounded by supporting cells and populate the sub-receptor zone. Synaptic terminals are not surrounded by supporting cells.

## Abbreviations

ABR: auditory brain stem response
AIS: axonal initial segment
AP: action potential
BK-channel: Big potassium or (calcium-activated potassium channel KCa1.1)
Caspr1: contactin-associated protein 1
CI: cochlear implant
CM: confocal microscopy
CNS: central nervous system
Cx30: connexin30
DAPI: 4′, 6-diamidino-2-phenylindole dihydro-chloride
diceCT: diffusible iodine-based contrast-enhanced computed tomography
EDTA: ethylene-diamine-tetra-acetic acid
EPSP: excitatory post-synaptic potential
GFAP: glia fibrillary acidic protein
GJ: gap junction
HCN: hyperpolarization-activated cyclic nucleotide-gated channels
HP: habenular perforata
IAC: internal acoustic canal
IGSB: intra-ganglionic spiral bundle
IHC: inner hair cell
Kv: voltage-gated potassium channel
LGC: large ganglion cell
LOC: lateral olive-cochlear
LW: lateral wall
MBP: myelin basic protein
MOC: medial olivo-cochlear
Micro-CT: micro-computerized tomography
Na/K-ATPase: sodium potassium adenosine-tri-phosphatase
Nav: voltage-gated sodium channel
NF: nerve fiber
NR: node of Ranvier
OC: organ of Corti
OHC: outer hair cell
PBS: phosphate buffered saline
PFD: paraformaldehyde
RC: Rosenthal's canal
RNA scope: ribonucleic acid detection device
RT-PCR: reverse transcription polymerase chain reaction
SEM: scanning electron microscopy
SG: spiral ganglion
SGCs: satellite glia cells
SGN: spiral ganglion neuron
SNHL: severe sensorineural hearing loss
SR-SIM: super-resolution structured illumination microscopy
TEM: transmission electron microscope/microscopy
TUJ1: tubulin-1
Type I cells: large spiral ganglion neurons (~95% of total numbers)
Type II cells: small spiral ganglion neurons (~5% of total numbers)
VGIC: voltage-gated ion channel
VGSC: voltage-gated sodium channel.
