# Supplementary figures and images for: Spike Generators and Cell Signaling in the Human Auditory Nerve: An Ultrastructural, Super-Resolution, and Gene Hybridization Study
Source: Front Cell Neurosci. 2021 Mar 16;15:642211. doi: 10.3389/fncel.2021.642211 (PMC8008129; doi:10.3389/fncel.2021.642211)

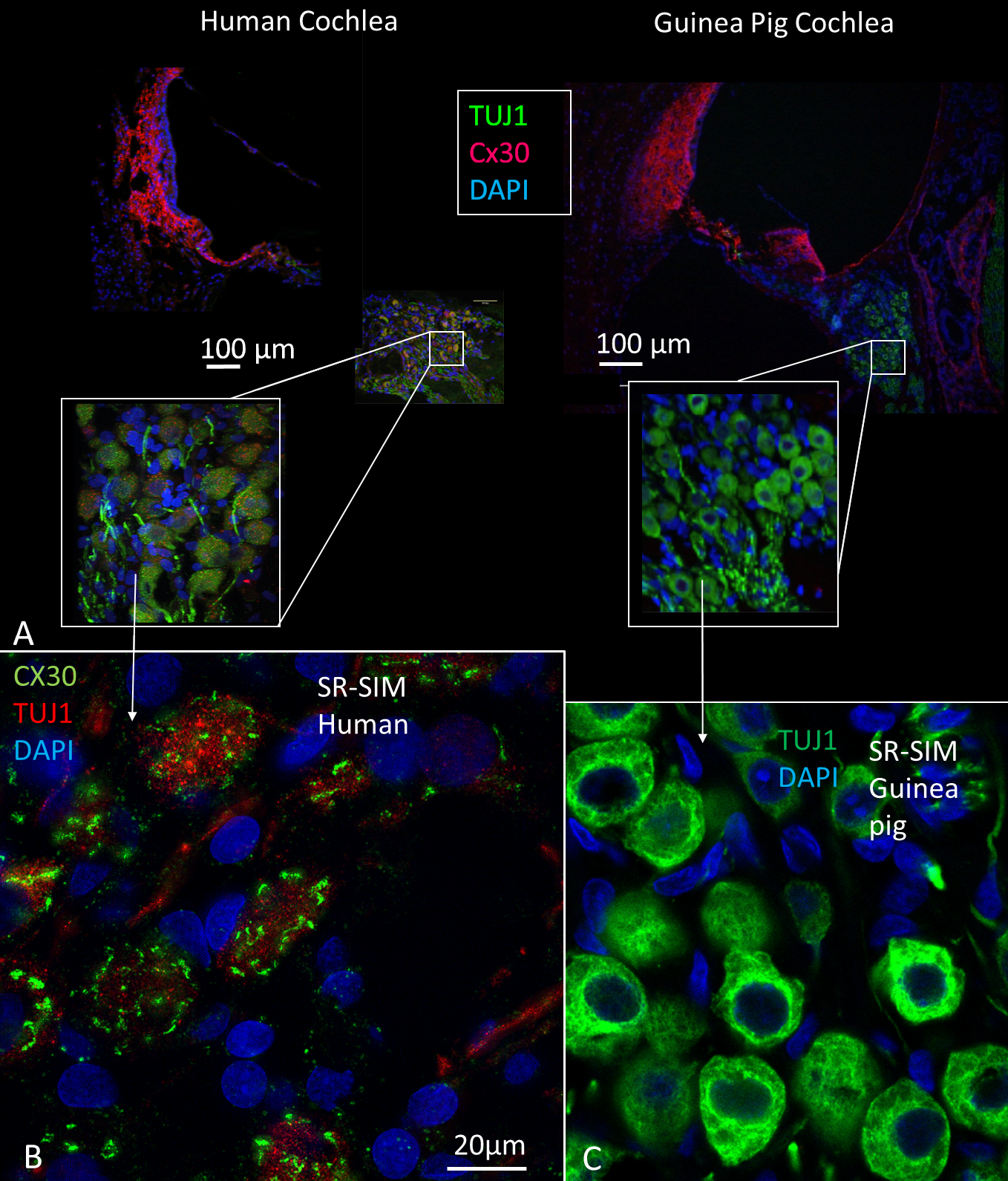

Supplement: Supplementary Figure 1 — (A) CM of Cx30 expression in the human and guinea pig cochlea. Cx30 is richly expressed in the lateral wall, OC, and spiral limbus in both species. Cx30 is expressed in human SGNs [framed area and (B)] but not in the guinea pig SGNs [framed area and (C)]. TUJ1; Neuron marker. DAPI; Cell nucleus marker (Supplementary Video 1). Cx30 is expressed in the human SGNs. [file Image_1.TIFF]

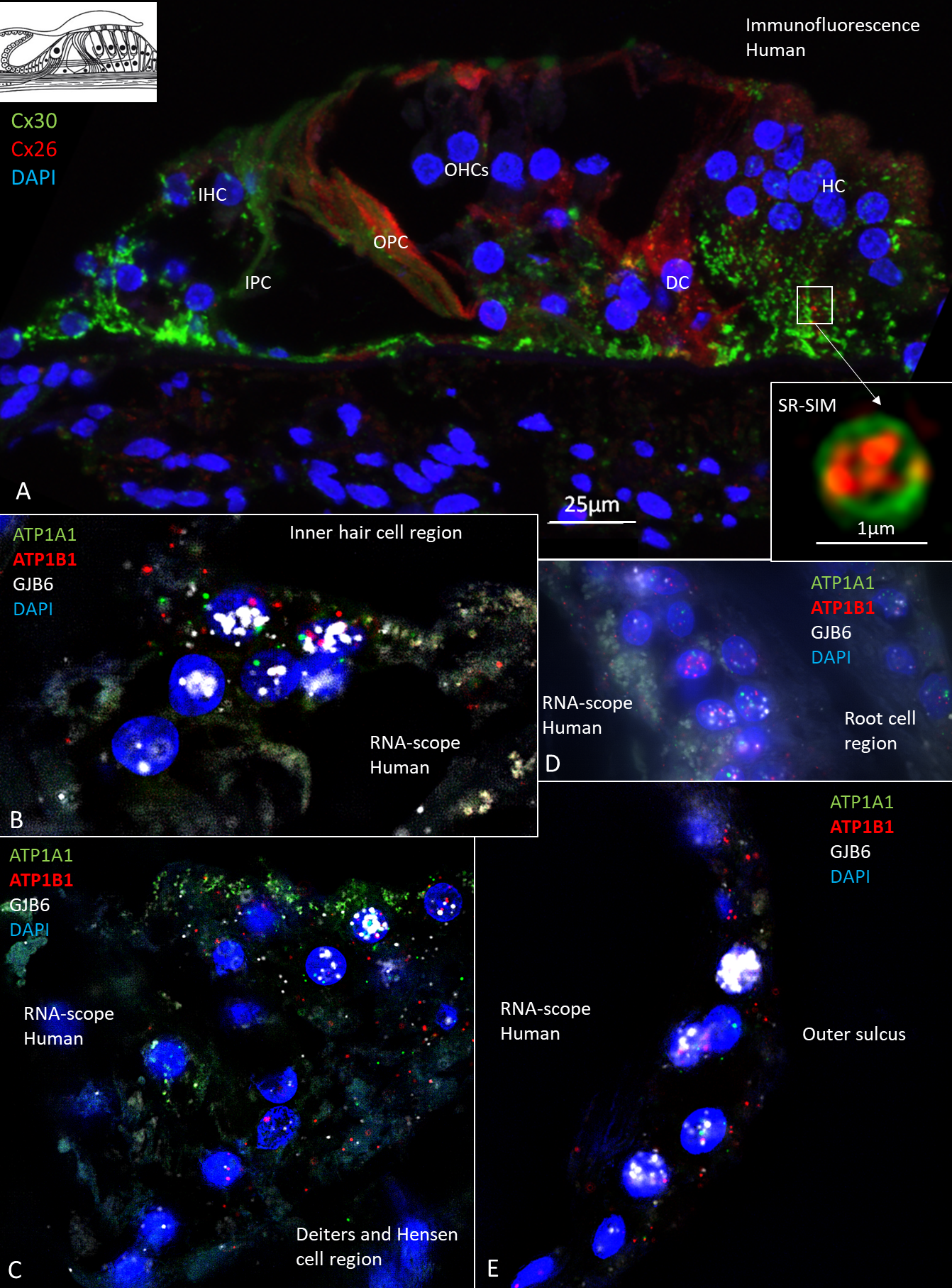

Supplement: Supplementary Figure 2 — (A) Immune fluorescence and SR-SIM (inset) show expression of Cx26 and Cx30 in the human OC. Cx30 is heavily expressed at the inner and outer hair cell region and among Hensen cells. SR-SIM shows that Cx26 and Cx30 are separately expressed RNAscope and four channel SR-SIM show localization of ATP1A1, ATP1B1 and GJB6 in the OC (B,C) and the lateral wall (D,E). Gene transcripts are expressed both in the cytoplasm and cell nuclei (Supplementary Video 2). 3D visualization of the CX26 and Cx30 expression shown in Figure 2A. [file Image_2.TIFF]

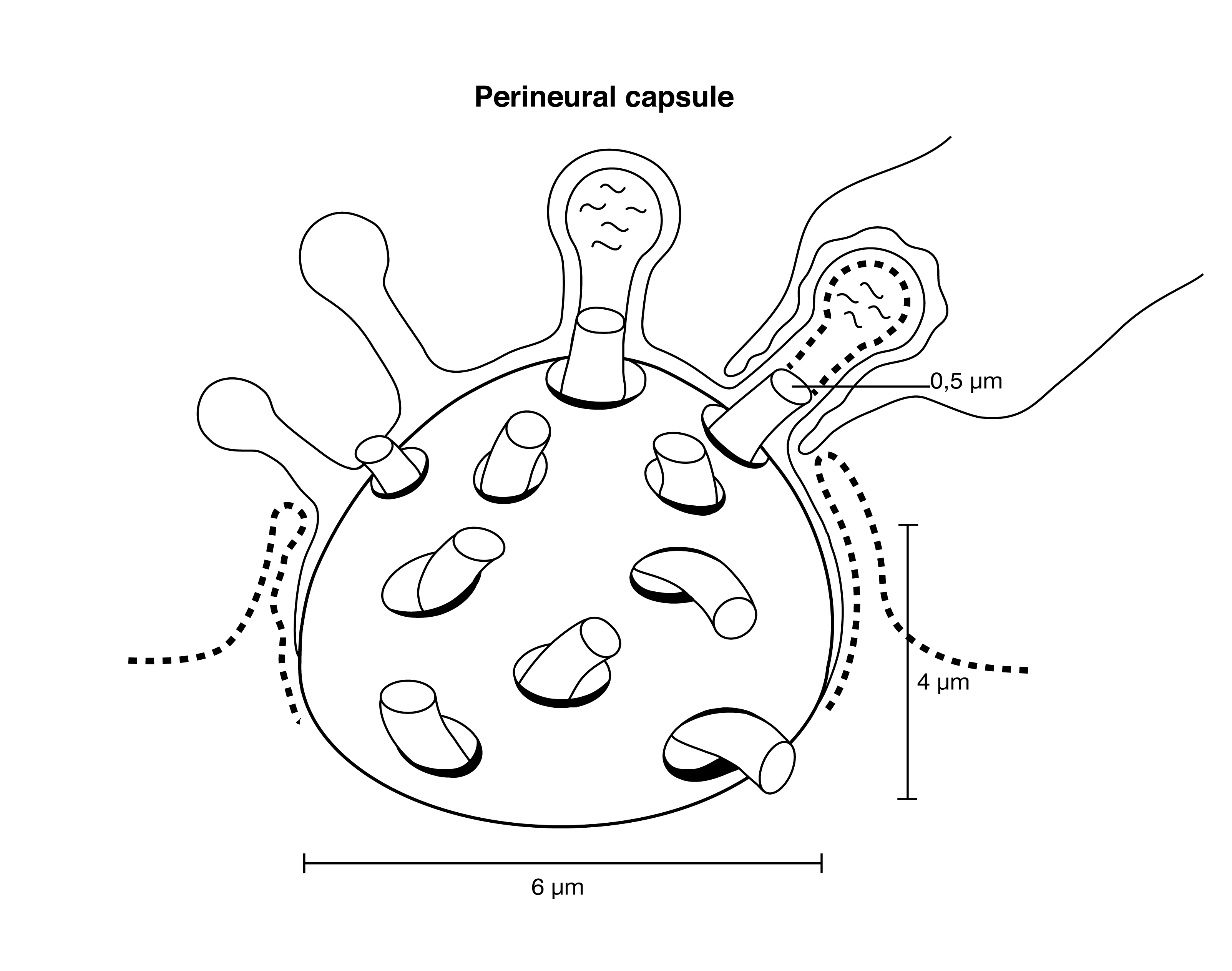

Supplement: Supplementary Figure 3 — Illustration of the human habenular canal opening into the organ of Corti based on SEM and TEM. Neurons are accompanied by thin glial cells from which they exit surrounded by supporting cells and populate the sub-receptor zone. Synaptic terminals are not surrounded by supporting cells. [file Image_3.jpeg]
